# Human regulator of telomere elongation helicase 1 (RTEL1) is required for the nuclear and cytoplasmic trafficking of pre-U2 RNA

**DOI:** 10.1093/nar/gku1402

**Published:** 2015-01-27

**Authors:** Michael Schertzer, Karina Jouravleva, Mylene Perderiset, Florent Dingli, Damarys Loew, Tangui Le Guen, Barbara Bardoni, Jean-Pierre de Villartay, Patrick Revy, Arturo Londoño-Vallejo

**Affiliations:** 1 Telomeres & Cancer laboratory, CNRS, ‘Labellisé Ligue’, Institut Curie, 26 rue d'Ulm, Paris 75248, France; 2 Sorbonne Universités, Paris 06, Paris F-75005, France; 3 PSL Research University, Paris, France; 4 Laboratory of Proteomic Mass Spectrometry, Institut Curie, Paris, France; 5 INSERM UMR 1163, Laboratory of Genome Dynamics in the Immune System, Paris, France; 6 Université Sorbonne Paris Cité, Université Paris Descartes, Institut Imagine, Paris, France; 7 IPMC-CNRS UMR7275–Valbonne, France; 8 Université de Nice Sophia-Antipolis, Nice 06560, France

## Abstract

Hoyeraal-Hreidarsson syndrome (HHS) is a severe form of Dyskeratosis congenita characterized by developmental defects, bone marrow failure and immunodeficiency and has been associated with telomere dysfunction. Recently, mutations in *Regulator of Telomere ELongation helicase 1 (RTEL1)*, a helicase first identified in *Mus musculus* as being responsible for the maintenance of long telomeres, have been identified in several HHS patients. Here we show that RTEL1 is required for the export and the correct cytoplasmic trafficking of the small nuclear (sn) RNA pre-U2, a component of the major spliceosome complex. RTEL1-HHS cells show abnormal subcellular partitioning of pre-U2, defects in the recycling of ribonucleotide proteins (RNP) in the cytoplasm and splicing defects. While most of these phenotypes can be suppressed by re-expressing the wild-type protein in RTEL1-HHS cells, expression of RTEL1 mutated variants in immortalized cells provokes cytoplasmic mislocalizations of pre-U2 and other RNP components, as well as splicing defects, thus phenocopying RTEL1-HHS cellular defects. Strikingly, expression of a cytoplasmic form of RTEL1 is sufficient to correct RNP mislocalizations both in RTEL1–HHS cells and in cells expressing nuclear mutated forms of RTEL1. This work unravels completely unanticipated roles for RTEL1 in RNP trafficking and strongly suggests that defects in RNP biogenesis pathways contribute to the pathology of HHS.

## INTRODUCTION

Regulator of Telomere ELongation helicase 1 (RTEL1) was first identified in *Mus musculus* as being responsible for the maintenance of long telomeres in embryonic stem (ES) cells (1). Mouse Rtel1 is involved in telomere replication, but also in genome wide replication, presumably by facilitating the progression of the replication fork (2,3). Mouse Rtel1 is also required for genome stability and repair (4) and the human protein has been suggested to restrict recombination through a helicase activity that dismantles recombination intermediate substrates *in vitro* (5,6). That human RTEL1 is involved in telomere metabolism has been recently confirmed by the identification of *RTEL1* mutations in patients with Hoyeraal-Hreidarsson syndrome (HHS), a severe form of dyskeratosis congenita characterized by short telomeres, developmental defects, bone marrow failure and immunodeficiency (7–11). However, the precise role of human RTEL1 remains largely speculative.

Based on results obtained from an initially unbiased analysis of potential human RTEL1 interactors, we set out to explore the role of this protein in non-coding RNA metabolism, both experimentally and in the context of HHS. We discovered that RTEL1 is required for the normal export of pre-U2 small nuclear (sn) RNA to the cytoplasm and for its trafficking through that compartment before being re-imported to the nucleus. U2 is a key component of the major spliceosome (12). It is transcribed by POLII as a precursor containing a 3′ extension (13). As soon as this snRNA is produced, its methylated cap is bound by the cap-binding complex, CBC (14), which is in turn bound by an activated (phosphorylated) form of phosphorylated adaptor for RNA export (PHAX) (15). PHAX is a mediator of snRNA export through its interaction with XPO1 (CRM1) (15). In the cytoplasm, the ribonucleotide protein (RNP) export complex dissociates and pre-U2 is transferred to the survival motor neuron (SMN) complex through its interaction with GEMIN5, another cap-binding protein (16). Once the pre-U2 snRNA is loaded onto the SMN complex, the assembly machine for spliceosomal RNPs (17), it undergoes maturation through both removal of the last 11–12 nucleotides and trimethylation of its cap, before being re-imported to the nucleus (17,18). Our results show that RTEL1 is required for the export of the pre-U2 RNP complex to the cytoplasm. Furthermore, mutations in the RING domain of the protein (13), carried by some HHS patients, are responsible for defects in the cytoplasmic trafficking of the pre-U2 RNP. Finally, we show that expression of mutated forms of RTEL1 impact the efficiency of splicing reactions, in agreement with a defect in U2 RNA biogenesis, and which strongly suggests that these defects may be responsible for at least some of the clinical manifestations and contribute to the severity of the disease phenotype in HHS patients.

## MATERIALS AND METHODS

### Cloning of RTEL1 cDNA and preparations of DNA constructs

For mammalian expression FL RTEL1 (nt 1–3903) was amplified from HeLa cells and was cloned as an EcoRI/HindIII fragment into pCMV-Tag2B (Stratagene), which contains an IRES-EGFP inserted as an XhoI/PacI fragment. The mutant RTEL1 ΔW (eliminating residues 34–48), and the ΔCter mutant (nt 1–3492) were created by polymerase chain reaction (PCR) and confirmed by sequencing (The sequences of primers used are available upon request). The RING domain was mutated by site directed mutagenesis using the QuickChange kit (Stratagene) and the primers top: 5′-TGACTTCCAGCGCGGCCAAGCCGGCTGGCAACGGCA.-3′, bottom: 5′-TGCCGTTGCCAGCCGGCTTGGCCGCGCTGGAAGTCA-3′; the amino acid modifications for the mutated RING (mR) are C1265A and C1268A. Mutating the NLS domain was done as follows: to generate the mutations R875A, K876A, K877A PCR was performed with the forward primer 5′-GCGGCCGCGATCCGGCTGGTCAGCCACCC-3′ and downstream primer, and with a reverse primer 5′-GCGGCCGCCCCTCCTCGCGGTTCTTCTGC-3′ and an upstream primer; in the process a NotI site was created which allowed cloning of the two fragments, which were then sub-cloned into pCMV-Tag2B containing RTEL1. For inducible expression the T-REX tetracycline system (Life Technologies) was used. The constructs were cloned as EcoRI/XhoI fragments into pcDNA-4/TO.

### Other expression plasmids used in this study

pGFP–NES and CY3–polydT20 was a gift from Valerie Doye.

### Anti-hRTEL1 antibody production

Custom rabbit antibodies against purified C-terminal RTEL1 (last 750aa) were prepared by AGRO-BIO. Immune sera from two rabbits were obtained and specific antibodies were affinity purified on NHS-activated sepharose columns covalently bound to C-terminal RTEL1. Antibody elution was carried out at acidic pH and antibody solutions were pH neutralized and stored at −20°C in 50% glycerol.

### Commercial antibodies

The following are other antibodies used in this study: anti-actin-HRP (horseradish peroxidase) (Santa Cruz, sc-47778), mouse anti-PCNA (Abcam, ab29), rabbit anti-XPO1-C-terminal (Santa Cruz, sc-5595), mouse anti-XPO1-N-terminal (Santa Cruz, sc-136220), mouse anti-Coilin (Sigma C-1862), mouse anti-XPO5 (Abcam ab57491), mouse anti-XPOT (Abcam ab49933), rabbit anti-H3 (Abcam ab1791), mouse anti-GEMIN5 (Santa Cruz, sc-136200), mouse anti-SMN1 (Abcam, ab5831), goat anti-LaminB (Santa Cruz, sc-6216), mouse anti-CSE1L (Santa Cruz sc-135855), rabbit anti-Dyskerin (Santa Cruz sc-48794), rabbit anti-PHAX (Bethyl, A303–916A), goat anti-PHAX (M-19) (Santa Cruz, sc-11704), mouse anti-UBF1 (Santa Cruz sc-13125), mouse anti-nucleolin (Enzo, ADI-KAM-CP100) mouse anti-TRF2 (Imgenex, IG124A), mouse anti-XPO5 (Abcam ab57491), mouse anti-NFKB (Santa Cruz sc-372), mouse anti-RANBP2 (Santa Cruz, sc-74518), mouse anti-Fibrillarin (Abcam, ab4566), mouse anti-FLAG M2 (Sigma), IgG mouse (Santa Cruz sc-2025), mouse anti-coilin (Sigma, C1862), rabbit anti-TCAB1 (Novus, NB100–68252); IgG rabbit (Abcam ab37415). All Alexa-conjugated secondary antibodies were purchased from Life Technologies.

### Cell lines and culture conditions

All cell lines were cultured in Dulbecco's modified Eagle's medium supplemented with L-glutamine, sodium pyruvate, non-essential amino acids (Life Technologies) and 10% fetal calf serum (Bio-West). 293T and Hela cells are from the Institute's collection. Hela cells stably expressing the Tet repressor were from Life Technologies. The 293T cell lines stably expressing RTEL1 were established by transfecting pCMV-FLAG- RTEL1-IRES-GFP into these cells followed by cell sorting (FACS Vantage, BD) three days later. A second, clonal sorting was performed 10 days later and lines stably over-expressing RTEL1 (as determined by western blot) were isolated. For inducible cell lines, Zeocin resistant cells were obtained and verified by western blot and immunofluorescence (IF).

### Patients’ fibroblast cells and controls

Fibroblasts were obtained from skin biopsies. SV40-transformed cell lines were obtained as previously described (19). Informed consent for our study was obtained from the families in accordance with the Helsinki Declaration. This study was also approved by the (INSERM) Institutional Review Board.

### siRNA mediated knockdowns

siRNAs against hRTEL1–1 (GCCUGUGUGUGGAGUAUGA), hRTEL1–2 (GACCAUCAGUGCUUACUAU), Luciferase (UCGAAGUAUUCCGCGUACG) or scrambled control were synthesized by Life Technologies. Knockdowns were performed using Amaxa (Lonza) solution R and program I-013. 200 pmoles of siRNA duplex was used and cells were left in culture 4–5 days before analysis.

### Transfections

Plasmid transfections were carried out using Effectene (Qiagen) according to the manufacturers instructions.

### Cell treatments

Leptomycin B, Actinomycin D and Cyclohexamide (Sigma) were used as indicated. All lysis buffers contained the anti-protease N-Ethylmaleimide (Sigma) at 10 mM.

### Western blot analyses

For fractionation, cells were treated with Cytoskeleton (CSK) buffer (0.5% Triton X-100; 10 mM, 1.4-piperazinediethanesulfonic acid (Pipes), pH 6.8; 100 mM NaCl; 300 mM sucrose; 3 mM MgCl2; 1 mM EGTA; 1mM PMSF) plus protease inhibitor cocktail (Roche). Cells were incubated on ice for 10 min, then centrifuged at 800 *g* for 5 min. The supernatant (soluble fraction) was separated and the pellet (insoluble fraction) was washed once in CSK and then resuspended in 2× sample buffer. Whole cell extracts were prepared by adding 2× sample buffer directly to cell pellets. Proteins were separated in 4–12% sodium dodecylsulphate-polyacrylamide gel electrophoresis (SDS-PAGE) gels (Life Technologies) and then transferred to PVDF membranes (GE Healthcare) for immunodetection. HRP-linked secondary antibodies (DAKO) were revealed using chemiluminescent detection (ECL Plus; GE Healthcare).

### Immunoprecipitations

Soluble lysates from either 293T, 293T stably overexpressing hRTEL1 or Hela cells, were prepared using NP40 lysis buffer (50 mM Tris–HCl pH 6.8, 150 mM NaCl, 1% NP-40, plus protease inhibitor cocktail). Approximately 500 μg of lysates were incubated with 10 μg of antibody or non-specific IgG. RNase A (Invitrogen) 0.1 μg/μl was added to indicated lysates for 30 min at 37°C. Complexes were bound to protein A or protein G agarose beads (Millipore). Beads were washed 3× with lysis buffer and 2× with phosphate buffered saline (PBS). Beads were resuspended in 100 μl of 2× sample buffer (0.125 M Tris–HCl, 4% SDS, 20% glycerol, 10% 2-mercaptoethanol, 0.004% bromphenol blue) and boiled. Proteins were analyzed by western blot.

### Liquid chromatography-MS/MS analysis

Soluble lysates from 1.8 × 10^8^ cells, 293T stably overexpressing hRTEL1 (ISO2) were prepared using lysing buffer (50 mM tris pH7.5, 300 mM NaCl, 10% glycerol, 10 mM Naf, 5 mM glycerophosphate, 5 mM Na pyrophosphate,1 mM B-mercaptoethanol, 0.1% NP40) incubated for 40 min at 4°C and homogenized by Dounce and then cleared by centrifugation at 10 000 *g* for 30 min. Lysat was incubated with rabbit anti-RTEL1 antibody covalently attached to protein A agarose beads or with rabbit preimmun antibody overnight at 4°C. Beads were washed 2× for 2 h with lysing buffer, and were resuspended in 200 μl of 1× sample buffer (60 mM Tris–HCl pH 6.8, 2% SDS, 10% glycerol, 5% 2-mercaptoethanol, 0.004% bromphenol blue). Eluted proteins were run on a 10% SDS-PAGE gel. In-gel digests were performed as described in standard protocols. Briefly, following the SDS-PAGE and washing of the excised gel slices proteins were reduced by adding 10 mM Dithiothreitol (DTT) (Sigma Aldrich) prior to alkylation with 55 mM iodoacetamide (Sigma Aldrich). After washing and shrinking of the gel pieces with 100% acetonitrile, trypsin (Sequencing Grade Modified, Roche Diagnostics) was added and proteins were digested overnight in 25 mM ammonium bicarbonate at 30°C. We achieved peptide concentration and separation using an actively split capillary HPLC system (Ultimate 3000 system, Dionex, Germering, Germany) connected to the LTQ Orbitrap XL™ mass spectrometer (Thermo SCIENTIFIC). The mass spectrometer was set to acquire a single MS scan followed by up to five data-dependent scan (dynamic exclusion repeat count of 1, repeat duration of 30 s, exclusion duration of 180 s and lock-mass option was enabled). The resulting spectra where then analyzed via the Mascot™ Software created with Proteome Discoverer (version: 1.2.0.92, Thermo Scientific) using the SwissProt Homo sapiens Protein Database (05 September 2012, 20 232 sequences). The resulting Mascot result files were loaded into the myProMS (20) server for further processing. In myProMS we fixed the estimated false discovery rate of all peptide and protein identifications to <1%, by automatically filtering on peptide length, mass error and Mascot score of all peptide identifications.

### Immunofluorescence

Cells grown on slides or cytospin preparations with 50 000 cells were fixed in Solution F (3% formaldehyde, 1× PBS, 300 mM sucrose) 15 min, when indicated after 5 min pre-extraction in Solution P (0.5% Triton-X-100, 20 mM Tris-HCl pH = 8.0, 50 mM NaCl, 5 mM MgCl_2_, 300 mM sucrose). Fixed cells were blocked with 10% horse serum in PBS, incubated sequentially in different primary antibodies, followed by fluorescently labeled secondary antibodies. All incubation steps were done in a humid incubator at 37°C for 1 h. Slides were mounted in Vectashield with 0.2 μg/ml 4′, 6-diamidino-2-phenylindole (DAPI). Images were taken with a 3D deconvolution microscope (Leica DM6000 B or Nikon) using the MetaMorph software. Final images are composed of arithmetic stacks of 20–30 deconvolved images, each 0.2 μm in depth.

### RNA FISH

Cells were washed in PBS, fixed for 10 min in 4% formaldehyde at RT; after fixation they were washed 2× in PBS for 5 min on ice. Cells were permeabilized with stepwise ethanol treatments 70–100%, then re-hydrated in 2× SSC, 25% formamide and pre-hybridized in 1×RHM (10% Dextran sulfate (Fluka), 2× SSC (Sigma), 2 mg/ml bovine serum albumin, 25% deionized formamide, 10 mM RVC in RNase-free water), 40 μg/ul tRNA (*Escherichia* *coli*, Roche), 1 μg/ul N50 (random oligo 50 bp), 1× Ribonucleoside Vanadyl Complex (NEB)) for 1 h at 37°C. Probes for four reactions were prepared as follows: 25 ng of biotinylated probe (pre-U2: CCGGAGGGGGTGCACCGTTCCT) (21); or CY3-polydT20) was prepared in hybridization buffer (40 μg tRNA, Roche, 1 μg N50, 25% formamide, 1× RHM, 1× RVC), heated and kept on ice. Slides were hybridized overnight at 37°C then washed 2× 20 min in formamide 25%, 2× SSC at 37°C and 1× in 2× SSC 5 min as described (21). For revelation, slides were washed at 37°C and then sequentially incubated with Avidin-FITC (1/400 in blocking solution); biotinylated goat-anti-Avidin (1/100 in blocking solution); Avidin-FITC (1/400 in blocking solution); and then washed. Slides were mounted with 0.5 ug/ul of DAPI and preparations were visualized using 3D deconvolution microscopy.

### RNA analysis

Total RNA was prepared using miRNeasy colums with DNase digestion (Qiagen). Fractionated lysates were also prepared according to the manufacturer's protocol (Qiagen). cDNAs were prepared with random hexanucleotide primers and the SuperScript III system according to the manufacturer's protocol (Life Technologies). The mRNA level was measured by real-time fluorescent quantitative RT-PCR and amplified using the GeneAmp 7500 sequence detection system and SYBR Green PCR Kits (Promega).

### RNA immunoprecipitations

RNA immunoprecipitations (IPs) were carried out following the protocol described by Keene *et al*. (22), with slight modifications. Fractionated lysates were obtained by treating cell pellets with buffer A (10 mM Tris–HCl pH 7.5, 10 mM NaCl, 3 mM MgCl_2_, 0.05% NP40) on ice for 10 min. The cytoplasmic fraction was retained after centrifugation. The pellet was washed 2× in buffer A and lysed in buffer B (50 mM Tris–HCl pH 6.8, 150 mM NaCl, 1% NP-40) on ice for 30 min and then cleared by centrifugation. Cytoplasm and nulceoplasm lysates were used for RNA-IPs as follows: approximately 500 μg of lysate were incubated with 10 μg of antibody or non-specific IgG for 4 h at 4°C. Complexes were incubated with A or G protein beads for 2 h at 4°C with agitation. After washing, RNA was eluted in 100 mM NaCl, 10 mM Tris–HCl pH 8.0, 10 mM Ethylenediaminetetraacetic acid pH 8.0, 1% SDS and treated with 200 μg proteinase K for 45 min at 50°C. Supernatants containing RNA were further using purified RNeasy columns (Qiagen) and cDNA was prepared as described above.

### Northern blot

Fractionated total RNAs were separated using a pre-cast 6% Tris/Borate/EDTA (TBE) Urea gel (Life Technologies). After electrophoresis, RNAs were transferred onto a nylon membrane and incubated with ^32^P-labeled oligonucleotides hybridizing to U snRNAs at 37°C in ExpressHyb solution (Clontech). Blots were rinsed 2× SSC 0.1% SDS briefly, 15 min at 37°C in 2× SSC 0.1% SDS and then exposed for 6 h to overnight in a phosphoimaging cassette (Molecular Dymanics). Images were developed using a STORM 860 phosphoimager (Molecular Dynamics). The oligonucleotides used were: preU2 (AACACGTTGTATCCCCGGTGG); U1 (CCCACTACCACAAATTATGC); U2 (TGGAGGTACTGCAATACCAGGT); U4 (GCCAATGCCGACTATATTGC); U6 (GCTTCACGAATTTGCGTGTCA); U12 (AAGTAGGCGGGTCACCTTG).

### Minigene splicing assay

Cells were transfected with the minicassette plasmid SXN13 (23) with Effectene (Qiagen). After 48 h, total RNA was extracted with miRNeasy columns (Qiagen) and cDNA prepared using oligo-dT and Superscript III (Life Technologies). PCR reactions to detect the splicing products were done using the primers SNXF: 5′-GACCATTCACCACATTGGTG-3′; and SXNRv: 5′-GAACCTCTGGGTCCAAGG-3′. PCR for specifically detecting partial splice products used SXNF and SXNJrv: 5′-GACCACCAGCAGCCTGGA-3′ or SKNJF: 5′-GCCCTGGGCAGGTCGAC-3′ and SXNRv. All products were electrophoresed in a 2% agarose gel.

*PCR Primers* (Table 1):

**Table 1.** PCR Primers

tRNA-TYR-F	CCTTCGATAGCTCAGCTGGTA;
tRNA-TYR-R	TCCTTCGAGCTGGAATCGAACC;
U2-F	ATACGTCCTCTATCCGAGGACA;
U2-R	TGGAGGTACTGCAATACCAGGT;
pre-U2-R	AACACGTTGTATCCCCGGTGG;
U1F	ATACTTACCTGGCAGGGGA;
preU1-R	GAATAACCCTTATAGGGGAGTC;
U4	GCTTTGCGCAGTGGCAGTA;
preU4-R	AAAATTCTCCGTAGAGACTGTCAA;
U12	TGCCTTAAACTTATGAGTAAGG;
preU12-R	ACCCCACAGTCAGTCTACTT;
SNHG3-F	CTCCTTGGATTTGTTAAGGATTC;
SNHG3-R	GTGAAAGAATGTGCATTCCTAGC
U97-F	CCCGATGATTATAAAAAGACGCG
U97-R	TTGCCCTCATATCTCATAATCTTC
WRN-F	CTCCCGTCAACTCAGATATGAGTA
WRN-R	TCAGAACCGGGAAAACATCTCCT
RAP1-F	ATAGCGGGGAACCACAGAATAAG
RAP1-R	ACCACAACCTCCTCAAACTCCC
MAPKAPK2-F	TGACCATCACCGAGTTTATGAAC
MAPKAPK2-R	AGAACCAAGGCAGAATTCAGTCA
ZNF45-F	CCACAGCTCTGTAGCTTCCTAG
ZNF45-R	TCCTACTTCTTGAAGAGTCTCC

### Statistical analyses

Pearson Chi2 tests were used for percentage comparisons.

## RESULTS

### RTEL1 localizes at the nucleolus and interacts with exportins

**Figure 1.**
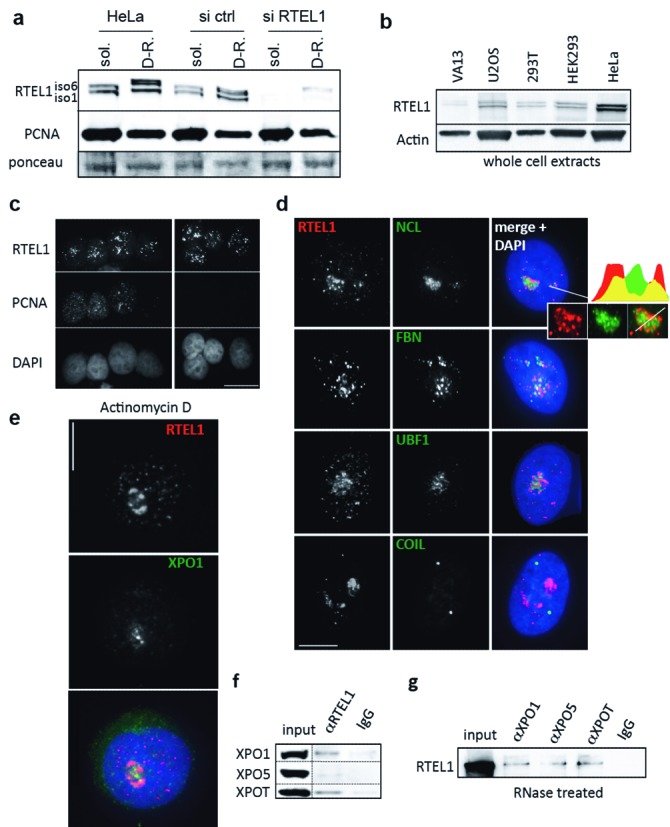
Human RTEL1 has a nucleolar localization and interacts with exportins. (**a**) Antibodies prepared against the C-terminus of the RTEL1_iso6_ protein specifically recognize both endogenous isoforms of RTEL1. Cells, unperturbed or transfected either with an irrelevant siRNA (si ctrl) or a siRNA against RTEL1, were lysed with CSK buffer and supernatants (soluble (sol.) proteins) and pellets (detergent-resistant (D-R) proteins) were separated in SDS-PAGE. PCNA and the ponceau staining of the membrane are shown as loading controls. (**b**) RTEL1 is expressed in all cells. Both isoforms are detected at variable levels in different types of human cell lines, as seen in western blots using whole cell extracts. (**c**) Immunofluorescence staining with anti-RTEL1 and anti-PCNA antibodies indicates that RTEL1 is mostly nuclear and expressed in S as well as in other phases of the cell cycle. Cells were pre-extracted, fixed with cold methanol and then fixed in PFA (scale bar 10 μm). (**d**) Reinforced signals of RTEL1 in the nucleus are found in around 20% of the cells associated with the nucleolus (mostly in the granular component) as indicated by co-localizations with three different nucleolar proteins: nucleolin (NCL), fibrillarin (FBN) and UBF1 (upstream binding factor 1). Coilin, a marker of Cajal bodies, does not colocalize with RTEL1. Cells were pre-extracted and fixed in PFA (scale bar 5 μm). (**e**) Actinomycin D treatment (50 ng/ml for 3 h) of cells leads to an enrichment of RTEL1 at the nucleolus in 100% of the cells. This response is similar to that observed with XPO1 (41). Cells were pre-extracted and fixed in PFA (scale bar 5 μm). (**f**) Validation of the interaction between RTEL1 and exportins in unperturbed HeLa cells (endogenous levels). Input represents 5% of total. (**g**) The RTEL1 interaction with exportins does not depend on the presence of RNA. Soluble protein extracts from 293T cells overexpressing RTEL1 were subjected to RNase treatment followed by IP using antibodies against XPO1, XPO5 and XPOT. Immunoprecipitates were analyzed with anti-RTEL1 antibody. Input represents 5% of total.

We have developed specific antibodies against RTEL1 (10), which revealed that both protein isoforms are associated with soluble as well as with detergent-resistant cell fractions, with a nuclear predominance and detectable in all phases of the cell cycle, as well as in all cell types tested (Figure 1a–c). IF experiments showed that, upon pre-extraction, RTEL1 displays a pattern of nuclear dots with a fraction of nuclei (≈20%) showing strong RTEL1 signals associated with nucleoli, particularly their granular component (Figure 1d). To further probe this association, we treated cells with actinomycin D (Act-D), an inhibitor of RNA polymerases I/II that provokes the redistribution of nucleolar components, with enrichment at this compartment of proteins with RNA-related functions (24,41). Upon Act-D treatment, RTEL1 was found associated with the nucleolus in 100% of cells (Figure 1e).

To identify RTEL1 partners and potential functions, we chose an unbiased approach and performed mass spectrometry analyses after RTEL1 IP using soluble extracts from 293T cells over-expressing RTEL1_iso6_, the longest isoform (10). These analyses revealed a large group of proteins involved in RNA metabolism, including the three major exportins XPO1, XPO5 and XPOT (Supplementary Table S1), all of which are involved in the nuclear export of ncRNAs. These interactions were validated in HeLa cells expressing endogenous levels of all proteins by co-IP using anti-RTEL1 antibodies (Figure 1f). Also, antibodies against each of the exportins were able to immunoprecipitate RTEL1 from RNase-treated 293T + RTEL1_iso6_ cell extracts, indicating that these interactions were RNA-independent (Figure 1g). Although the interactions of RTEL1 with exportins, as revealed by these IP experiments, appear to involve only a small fraction of both partners, they are likely specific since antibodies against XPO1, which failed to recognize RTEL1, even when overexpressed (Supplementary Figure S1a), were able to immunoprecipitate only a small fraction of PHAX (Supplementary Figure S1b), the dedicated partner of XPO1 for snRNA trafficking and export (15). Furthermore, IF analyses showed that, in unperturbed HeLa cells, RTEL1 and all three exportins are always found in very close proximity to each other and close to the nucleolus (Supplementary Figure S1c–e). These co-localizations are in contrast to the lack of co-localization between RTEL1 and TRF2 (Supplementary Figure S1f), a major component of the protein telomere complex and which was never found in our mass spectrometry analyses.

### RTEL1 is part of the pre-U2 RNP complex and is required for the intranuclear trafficking and cytoplasmic export of pre-U2

**Figure 2.**
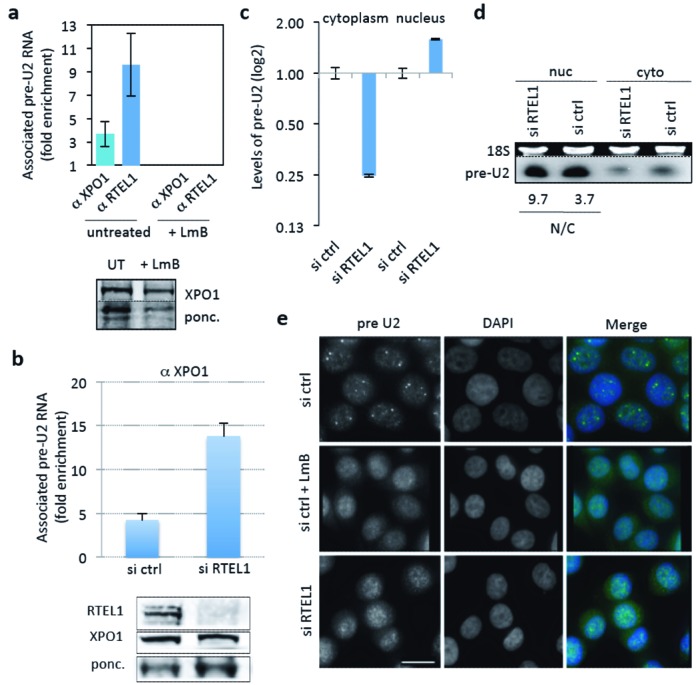
RTEL1 is required for nuclear trafficking and export of pre-U2 to the cytoplasm. (**a**) The interaction of RTEL1 with pre-U2 is XPO1-dependent. Antibodies against XPO1 and RTEL1 were used to assess the association of these proteins with pre-U2 by RNA-IP. Antibodies against RTEL1 very efficiently immunoprecipitated pre-U2 molecules (as measured by RT-qPCR) indicating that RTEL1 is part of the pre-U2 RNP complex. This interaction, as well as that of XPO1 with pre-U2, is completely lost in cells treated with LmB, indicating that RTEL1 binds to the RNP complex through XPO1 (shown is the fold enrichment compared to control IgG). Below the histogram is a western blot showing anti-XPO1 IP efficiency in untreated and LmB-treated cells. Input represents 5% of total. (**b**) siRNA-mediated depletion of RTEL1 does not alter the formation of the XPO1–RNP complex, as shown by RNA-IP using anti-XPO1 antibody and measuring the levels of pre-U2 (shown is the fold enrichment compared to control IgG). XPO1 protein levels in the cell remain unchanged upon RTEL1 depletion (bottom panel). Whole-cell protein extracts were used for western blot analysis and IP 5 days after siRNA RTEL1 transfection. (**c**) RTEL1 is required for the export of pre-U2. The concentrations of pre-U2 in the nucleus and the cytoplasm were determined by RT-qPCR using nuclear and cytoplamic extracts prepared from si ctrl- and si RTEL1-transfected HeLa cells. The levels were normalized to an unaffected RNA (SNHG3) and then normalized to that ratio in the control. In the absence of RTEL1 pre-U2 becomes enriched in the nucleus and depleted in the cytoplasm. (**d**) The relative decrease in the concentration of cytoplasmic versus nuclear pre-U2 in RTEL1 KD cells is confirmed by northern blot analysis. The EtBr-stained gel before transfer (upper panel) is shown for loading control. The numbers below represent the ratio of nuclear versus cytoplasmic pre-U2 (calculated after normalization for loading). There is relatively more RNA in the nuclear compartment of si RTEL1-treated cells than in cells treated with si ctrl. (**e**) RNA FISH experiments using a specific probe against pre-U2 show a nucleoplasmic localization of this RNA with a few stronger foci (presumably Cajal bodies) in HeLa cells transfected with an si RNA control. Treatment of the same cells with the XPO1-specific inhibitor LmB leads to the elimination of such nuclear foci. The same phenotype is detected in RTEL1-depleted cells, indicating that RTEL1 is also required for pre-U2 intranuclear trafficking (scale bar 10 μm).

We decided to explore in more detail the interaction between RTEL1 and XPO1. XPO1 is critical for the intranuclear trafficking and cytoplasmic export of some spliceosomal snRNAs, the best characterized being the precursor of snRNA U2 (pre-U2), a component of the major spliceosome. Given that RTEL1 seemed to interact with XPO1 in an RNA independent manner, we wondered whether RTEL1 was able to interact with XPO1 when the latter is part of the pre-U2 RNP export complex. Indeed, antibodies against RTEL1 were able to efficiently immunoprecipitate pre-U2 molecules (Figure 2a). This RTEL1/pre-U2 interaction was completely lost in cells treated with Leptomycin B (LmB), an inhibitor of XPO1 activities (25), indicating that RTEL1 does not bind directly to the RNA but instead entirely depends on XPO1 for its binding to the pre-U2 RNP complex (Figure 2a). To at least partially assess the extent of RTEL1 interactions with other exported U snRNAs, we tested whether anti-RTEL1 antibodies were able to immunoprecipitate U1 and U4 (two other components of the major spliceosome, which are also exported to the cytoplasm in a XPO1-dependent manner (26–28)), as well as U12, the equivalent of U2 for the minor form of spliceosomes and which is expected to follow similar maturation pathways (29,30). As shown in Supplementary Figure S2a, all three U snRNAs were significantly enriched after RTEL1 RNA-IP, although at different levels.

The fact that RTEL1 interacts with exportins, and in particular the pre-U2 RNP complex, suggested that RTEL1 is a factor for nuclear trafficking, cytoplasmic export or both. Upon transcription, the pre-U2 RNA is bound by the CBC (31), which in turn is bound by PHAX (15), an adaptor molecule containing a nuclear export signal (NES). This NES domain is recognized by XPO1 thus allowing the formation of the pre-U2 RNP complex, which is then exported to the cytoplasm (18). While the XPO1 interaction with pre-U2 did not depend on RTEL1 (Figure 2b), the nucleus/cytoplasm partition of pre-U2 RNA molecules was altered in RTEL1-depleted cells, as shown both by RT-qPCR and by northern blot (Figure 2c and d). Specifically, we observed an increase in nuclear pre-U2 in comparison with cytoplasmic pre-U2 in cells depleted for RTEL1, as shown both by RT-qPCR and by northern blot (Figure 2c, d and Supplementary Figure S2b). The finding that the levels of pre-U2-XPO1 RNP increase in the absence of RTEL1, while confirming that the biogenesis of this RNP does not depend on RTEL1, is consistent with a failure of the complex to be exported, since once in the cytoplasm, the complex immediately dissociates (16). On the other hand, RNA-FISH experiments using a specific pre-U2 probe revealed that, upon RTEL1 depletion, the pre-U2 RNA no longer accumulated at discrete nuclear foci (previously reported to be Cajal bodies and sites of pre-U2 processing (32) and whose formation is dependent on XPO1 (21)), but instead adopted a diffuse pattern identical to that observed upon treatment of cells with LmB (Figure 2e). Of note, depletion of RTEL1 did not prevent the formation of Cajal bodies in the same cells (Supplementary Figure S2c).

The above experiments establish that RTEL1 interacts with and is required for the correct intranuclear trafficking and cytoplasmic export of pre-U2 RNP. In contrast, RTEL1 is not required for mRNA export, since the depletion of RTEL1 has no effect either on the overall cytoplasmic/nuclear partitioning of poly-A mRNAs, as determined by RNA-FISH (Supplementary Figure S2d) or on the cytoplasmic/nuclear partition of specific mRNAs, as measured by qRT-PCR (Supplementary Figure S2e). Importantly, the export to the cytoplasm of reporter proteins carrying an NES is not perturbed upon RTEL1 KDs (Supplementary Figure S2f), indicating that RTEL1 is not involved in the XPO1-dependent, NES-mediated pathway of protein export.

### HHS patients carrying *RTEL1* mutations display abnormal pre-U2 cell distributions

**Figure 3.**
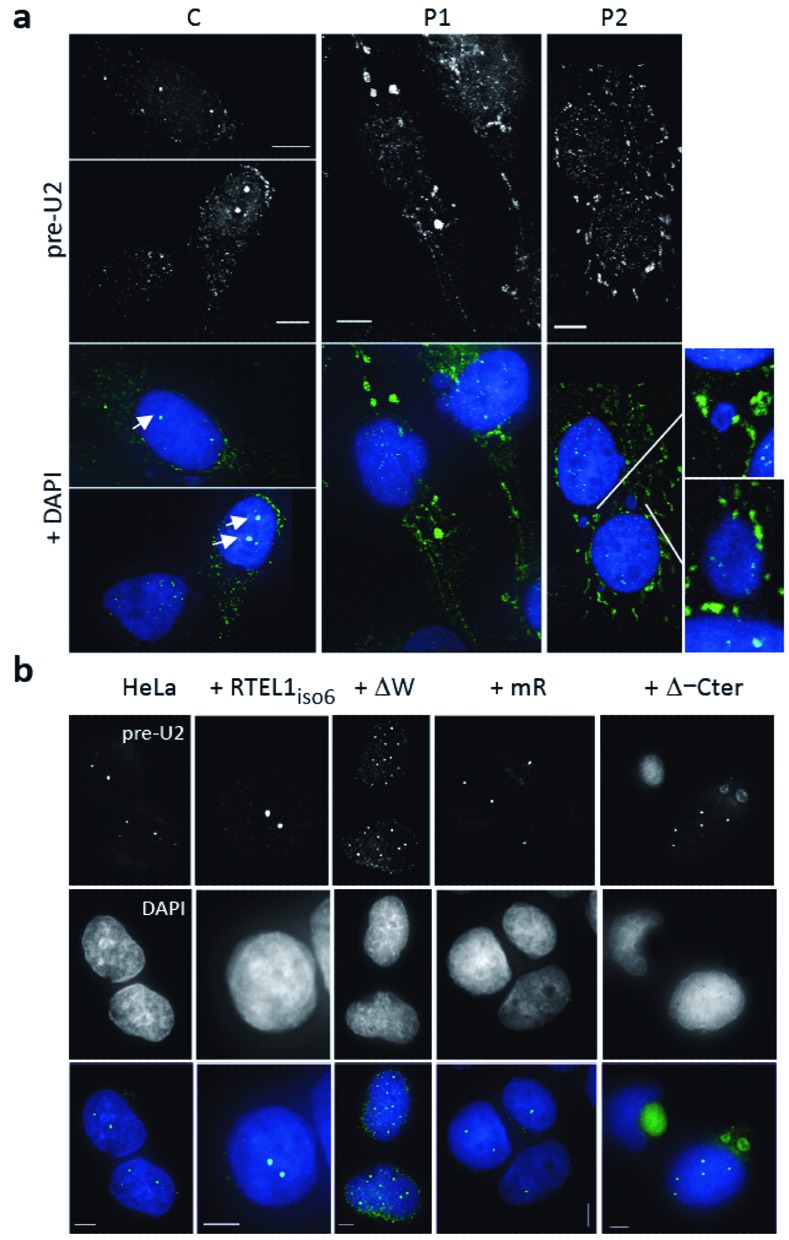
Both RTEL1-HHS cells and cells expressing mutated RTEL1 variants display defects in pre-U2 trafficking through the cytoplasm. (**a**) Pre-U2 accumulates in the cytoplasm of cells from HHS patients carrying *RTEL1* mutations. Specific pre-U2 signals are revealed by RNA-FISH (in white above/in green below). Arrows point to strong nuclear foci observed in control cells (C) and that are infrequently observed in RTEL1 patient cells (P1 and P2) (see Supplementary Figure S3a for a quantification). Scale bar 5 μm. (**b**) Expression of mutated forms of RTEL1 lead to pre-U2 mislocalization in the cytoplasm. Coarse or granular mislocalizations of pre-U2 (in green) are detected by RNA FISH in the cytoplasm of HeLa cells expressing RTEL1 variants carrying mutations in the Walker domain (ΔW) or, more evidently, when missing the last C-terminal domain (Δ-Cter) (scale bar 5 μm).

We next examined the pre-U2 status in immortalized fibroblasts obtained from HHS patients with mutations in RTEL1. Strikingly, the lack of nuclear focus formation by pre-U2 observed in cells experimentally depleted for RTEL1 was also a characteristic of cells from RTEL1–HHS patients, where, in addition, pre-U2 molecules were found to accumulate in large cytoplasmic structures (Figure 3a and Supplementary Figure S3a). Of note, these large cytoplasmic accumulations were also experimentally provoked, in HeLa cells, by the induced expression of mutated versions of RTEL1. Specifically, a version carrying a deletion in the conserved helicase Walker A box domain and another mutant missing the last 150 amino acids of the protein (called hereafter Δ-Cter) (Figure 3b). The fact that large cytoplasmic accumulations of pre-U2 RNAs were detected in RTEL1 patients’ cells (which express at least one mutated RTEL1 isoform, see Supplementary Figure S3b) and upon forced expression of abnormal RTEL1 versions, suggests that pre-U2 can be exported in the presence of mutated forms of RTEL1; nevertheless, in such a context the exported molecules appear to be unable to accomplish their normal cytoplasmic itinerary and therefore accumulate in that compartment.

### RTEL1 is required for the correct cytoplasmic trafficking of RNP components

Immediately after export, cytoplasmic pre-U2 RNA dissociates from the XPO1–PHAX–CBC complex in a multistep process that is not completely understood (18). The unloading of the RNA from the exported RNP is necessary for the loading of GEMIN5, which binds the methylated cap as well as internal sequences (16,33). This association promotes the recruitment of SMN1, which in turn fosters the formation of the full Sm core complex thus triggering the trimethylation of the guanosine cap, the 3′-end trimming of the RNA and the re-importation of the matured U2 into the nucleus (16,18).

**Figure 4.**
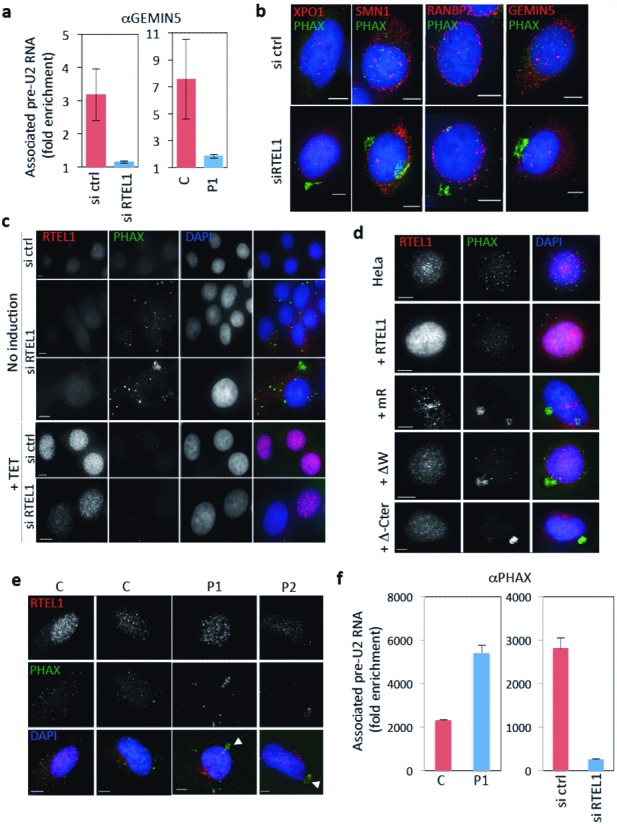
Dysfunctional RTEL1 leads to defects in the traffic of RNP components though the cytoplasm. (**a**) RNA-IPs using cytoplasmic lysates and anti-GEMIN5 antibodies to detect its interaction with pre-U2 show a deficiency in the formation of the cytoplasmic complex both in RTEL1-depleted cells (left) and in RTEL1-HHS patients (right) (C: control cells; P1: Patient's cells). Cells were treated with cyclohexamide. Levels are expressed as fold enrichment compared to control IgG. Western blot (whole cell extracts) illustrating the level of RTEL1 depletion is shown in Supplementary Figure S4a. (**b**) IF experiments using antibodies against PHAX (in green) shows abnormal accumulation of the protein in the cytoplasm of cells depleted for RTEL1. The localization of other proteins such as XPO1, SMN1, GEMIN5 or RANBP2 (all in red) is not perturbed. Cell preparations were fixed in PFA without pre-extraction (scale bar 5 μm). (**c**) Induction of expression of RTEL1_iso6_ in siRNA RTEL1-treated cells counteracts the accumulation of PHAX in the cytoplasm indicating that the phenotype is due to RTEL1 depletion. HeLa cells carrying a TET-inducible cassette controlling the expression of WT RTEL1_iso6_ were transfected with the siRNA RTEL1 or siRNA ctrl and tetracycline was added or not after 4 days for 24 h. Cell preparations were fixed in PFA without pre-extraction (scale bar 5 μm). Western blots (whole cell extracts) confirming the levels of depletion and induction are shown in Supplementary Figure S4b. A quantification of this experiment is shown in Supplementary Figure S4d. (**d**) Coarse accumulations of PHAX (in green) are also detected in HeLa cells induced for the expression of RTEL1 variants carrying mutations in the Walker domain (ΔW), the RING domain (mR) or missing the last C-terminal domain (Δ-Cter). A quantification of these data is presented in Supplementary Figure S4e. RTEL1 is shown in red (scale bar 5 μm). Cell preparations were fixed in PFA without pre-extraction. (**e**) Cells from HHS patients (P1 and P2) carrying *RTEL1* mutations also show coarse accumulations of PHAX (shown in green, arrows) in the cytoplasm. Cell preparations were fixed in PFA without pre-extraction (scale bar 5 μm). Quantification of these data is presented in Supplementary Figure S4g. (**f**) Pre-U2 RNA IPs using anti-PHAX antibodies in cytoplasmic lysates. In RTEL1-HHS patients’ cells (P1), the levels of pre-U2–PHAX complex detected in the cytoplasm is increased, compared to the control fibroblasts (C, left histogram), whereas the levels of this complex is decreased in RTEL1-depleted HeLa cells, as expected from the export defect, compared to control (right histogram). Values represent fold enrichment compared to IgG control.

We examined the consequences of the RTEL1 dysfunction on pre-U2 cytoplasmic trafficking by following the association of this molecule with GEMIN5. To increase the sensitivity of GEMIN5 RNA-IPs, we treated cells with cyclohexamide, which dissociates GEMIN5 from the Sm core complex and allows the detection of transient GEMIN5–RNA intermediates before delivery to SMN1 (16). As expected because of the observed export defect, depletion of RTEL1 in HeLa cells led to a decrease in the amount of pre-U2 that was associated with GEMIN5 in the cytoplasm (Figure 4a and Supplementary Figure S4a). Intriguingly, a similar decrease was detected in the RTEL1–HHS cells (Figure 4a), in spite of the strong pre-U2 accumulation in the cytoplasm (Figure 3a), suggesting that an intact RTEL1 protein is also required for the correct transfer of the pre-U2 molecule to GEMIN 5 after nuclear export.

Since the transfer of pre-U2 onto GEMIN5, which is another 7-methylguanosine cap-binding protein (33), requires the unloading of CBC/PHAX from the RNA, we wished to assess the status of cytoplasmic PHAX in cells mutated for RTEL1. Depleting cells for RTEL1 led to a clearly visible abnormal accumulation of PHAX in large cytoplasmic, DAPI-negative, paranuclear foci, without perturbing the distributions of XPO1, RANBP2, GEMIN5 or SMN1 (Figure 4b). Similar cytoplasmic accumulations of PHAX have been described upon perturbation of the processing of pre-U2 by introduction of a dominant negative form of SMN1 (34). Interestingly, the expression of WT RTEL1_iso6_ (but not that of RTEL1_iso6_ carrying mutations in the RING domain) rescued the PHAX phenotype (Figure 4c, Supplementary Figure S4b–d) in cells depleted for RTEL1, thus providing direct evidence for the RTEL1 involvement in the recycling of this critical component of the RNA export pathway. These experiments also revealed the requirement of an intact RING domain for this particular function. In addition, the same aberrant PHAX phenotype was observed both experimentally upon expression of mutated RTEL1 alleles, including RTEL1_iso6_ mR (Figure 4d and Supplementary Figure S4e) and in RTEL1-HHS cells (Figure 4f). Of note, PHAX cytoplasmic accumulations in RTEL1-HHS cells were rescued by the exogenous expression of wild-type RTEL1_iso6_ (Supplementary Figure S4f and g), thus reinforcing the evidence that RTEL1 mutations in HHS patients are directly responsible for the phenotype. In addition, RNA-IP experiments using anti-PHAX antibodies revealed an increased level of association of PHAX with pre-U2 in the cytoplasm in HHS patients’ cells (Figure 4f), thus lending support to the idea that, upon export, there is a failure to transfer the pre-U2 RNA from the CBC–PHAX–RNP to GEMIN5. On the contrary, and as expected from the export defect, cells depleted for RTEL1 show a lower level of PHAX–pre-U2 association in the cytoplasm (Figure 4f). Together, these experiments indicate that in the presence of a dysfunctional RTEL1 protein, a failure in the transfer of pre-U2 RNA to GEMIN5 occurs, and the defect is associated with a default in the recycling of the exported RNP component PHAX.

### A cytoplasmic form of RTEL1 rescues the cytoplasmic defects in RNP trafficking induced by RTEL1 depletion or by expression of mutated nuclear RTEL1

Two alternative hypotheses can be envisioned to explain the effects seen upon expression of mutated forms of RTEL1: either these proteins promote the export of an abnormal RNP, which is then not appropriately handled in the cytoplasm or RTEL1 is directly required in the cytoplasmic compartment to help in the pre-U2 transfer. Indeed, the experiments described above suggest that potential RTEL1-dependent cytoplasmic activities may take place between the disassembly of the CBC-PHAX-RNP and the transfer of pre-U2 to GEMIN5.

**Figure 5.**
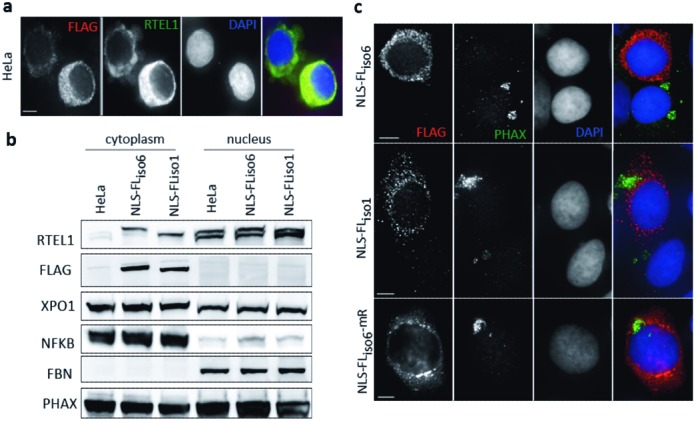
A crucial role of RTEL1 in RNP trafficking occurs in the cytoplasm. Cytoplasmic versions of RTEL1 were created by mutating the NLS consensus sequence. All NLS-null proteins were flag-tagged allowing its specific detection in IFs (**a**) and western blots (**b**). Both approaches confirmed that the NLS null protein remains cytoplasmic. For IFs, cell preparations were fixed in PFA without pre-extraction (scale bar 5 μm). For Western blot (WB), cytoplasmic proteins were extracted with CSK buffer. Nuclear proteins were further extracted in the presence of 600 mM NaCl. Compartmentalization was verified by revealing with antibodies against NFKB (cytoplasmic) and fibrillarin (FBN) (nuclear). (**c**) An NLS mutated RTEL1_iso6_ can rescue the cytoplasmic phenotypes induced by a mutated nuclear form of RTEL1. HeLa cells induced for the expression of RTEL1_iso6_-mR accumulate PHAX in the cytoplasm. Exogenous expression of NLS-null RTEL1_iso6_ is able to eliminate this phenotype. However, NLS-null RTEL1_iso6_-mR or RTEL1_iso1_ (which naturally lacks the RING domain) are not capable of doing this, indicating that the RING domain is crucial for this cytoplasmic activity. Quantitative data can be found in Supplementary Figure S5b. Cell preparations were fixed in PFA without pre-extraction (scale bar 5 μm).

The RTEL1 protein carries a unique putative nuclear localization signal (NLS, aa 871–877) immediately after the helicase domain and right before the recently identified Harmonin-like domains in the C-terminus (35). Although a small fraction of RTEL1 can be detected in the cytoplasm, most of the protein is detected in the nucleus (Figure 1). To test whether or not RTEL1 has a cytoplasmic role, we designed rescue experiments of cytoplasmic PHAX defects using a version of RTEL1 that is confined to the cytoplasmic compartment. We mutated the predicted NLS in RTEL1_iso6_ and expressed an N-terminal FLAG tagged version of this protein exogenously. As expected, the protein was found to accumulate exclusively in the cytoplasm, both by IF and by western blot analyses (Figure 5a and b). Expression of an NLS null RTEL1_iso6_ protein in cells where expression of RTEL1_iso6_ -mR had been induced, completely corrected the PHAX cytoplasmic accumulations (Figure 5c). Interestingly, an NLS mutated version of the naturally occurring shorter form of RTEL1 (RTEL1_iso1_, which lacks the RING domain) was unable to rescue the PHAX phenotype (Figure 5c). This observation, and the fact that an NLS-null RTEL1_iso6_-mR version was also unable to rescue the PHAX phenotype (Figure 5c and Supplementary Figure S5a), supports our contention that the RING domain in RTEL1 is necessary for the correct resolution of cytoplasmic PHAX intermediates. Finally, NLS null RTEL1 proteins induced strong cytoplasmic accumulations of pre-U2 in control cells (Supplementary Figure S5b and c), suggesting that while nuclear import of RTEL1 is dispensable for PHAX nuclear reintegration, it is necessary for a correct maturation of pre-U2 and, likely, nuclear import of U2.

### Splicing defects in cells expressing mutated RTEL1 and in HHS cells

**Figure 6.**
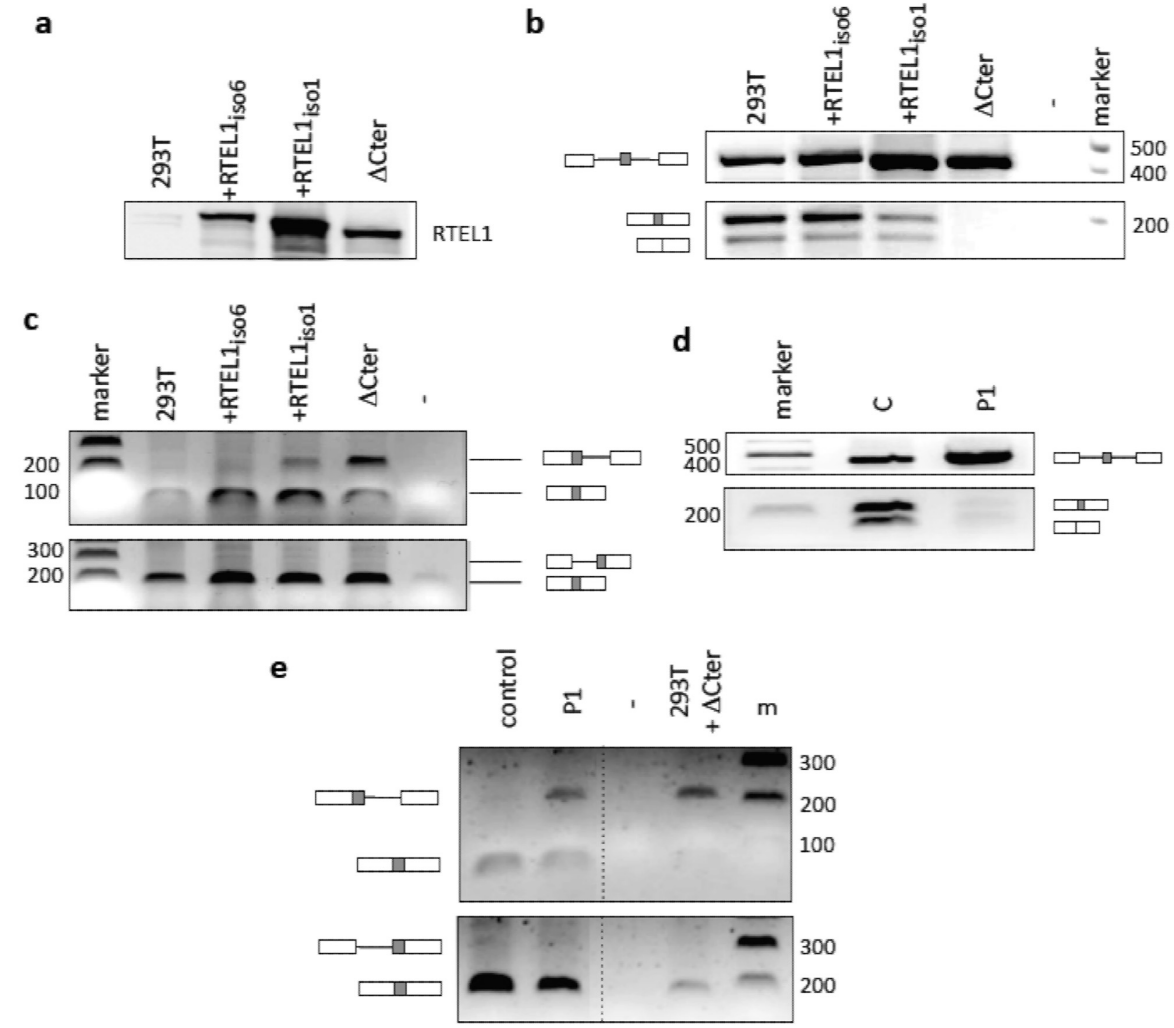
RTEL1 deficiency leads to splicing defects. (**a**) Western blot showing levels of expression of exogenous RTEL1 proteins in 293T cells (equivalent amounts of whole cell extracts). (**b**) Impact of such expressions on the splicing efficiency of introns flanking an exon minicassette. Unperturbed 293T cells, or 293T cells expressing exogenous RTEL1 proteins were transfected with the minicassette plasmid. After 48 h, RNA was extracted, cDNA prepared and PCR reactions using the indicated primers (Supplementary Figure S6a) were conducted. Shown are the bands corresponding to the indicated products (top: unspliced primary transcript, middle: splicing of both introns, bottom: skipping of the middle exon). Cells expressing the C-terminus deleted RTEL1 protein are unable to carry out these reactions efficiently. (**c**) Accumulation of partially spliced species in 293T cells expressing the C-terminus deleted RTEL1 protein. A different PCR approach using junction primers (Figure 6b) allowed the detection of partially spliced products that failed to carry out the second splicing reaction on the same substrate (upper panel). No partially spliced products conserving the first intron are detected (bottom panel). (**d**) Splicing deficiencies are also detected in patients’ cells (P1) carrying *RTEL1* mutations. (**e**) These deficiencies are also due to the inability to efficiently achieve the second splicing event.

Given that cells expressing mutated forms of RTEL1 lead to alterations in the biogenesis of U2, a critical component of the major spliceosome, it would be expected that such cells will display splicing defects. Using a previously described (23) exogenous mini-gene splicing assay (Supplementary Figure S6a), we examined the efficiency of the splicing reaction in 293T cells stably over-expressing a wild-type form of the RTEL1 protein (either isoform 1 or 6) or overexpressing the Δ-Cter mutated form (Figure 6a), shown above to induce dramatic perturbations in the cytoplasmic traffic of pre-U2 RNP. We found that expression of this particular RTEL1 mutant, but not that of the wild-type form, showed a reduced splicing efficiency of the mini-gene and also the accumulation of partially spliced molecules (Figure 6b). A PCR-based approach (Supplementary Figure S6b), allowed us to determine that such partially spliced molecules retained the second intron (Figure 6c), thus revealing a specific failure in the second splicing reaction. Importantly, HHS cells also displayed similar splicing defects using the same splicing assay (Figure 6d and e). These experiments allow us to confirm that the effects of RTEL1 mutations on U2 biogenesis have a biological impact, notably on splicing.

Finally, since RTEL1 interacts with other POLII-dependent U snRNAs (shown in Supplementary Figure S2a) that follow similar PHAX-dependent export and SMN-dependent cytoplasmic maturation pathways (26,36), we wished to determine whether the effect of RTEL1 mutations on splicing efficiency was exclusively explained by its impact on U2 biogenesis. In fact, northern blot analyses revealed that not only the levels of U2 but those of U1 and U12 appeared to be reduced in cells overexpressing RTEL1-Δ-Cter (Supplementary Figure S6c and d), supporting the idea that RTEL1 dysfunction impacts spliceosome activities through perturbation of U snRNA components. Furthermore, RNA IPs using nuclear lysates and PHAX antibodies showed an increased interaction of this adaptor with pre-U1 and pre-U12 in HeLa cells depleted for RTEL1 (Supplementary Figure S6e). This last observation is consistent with the idea that in the absence of RTEL1 there is a generalized defect in the trafficking and export of U snRNPs.

## DISCUSSION

**Figure 7.**
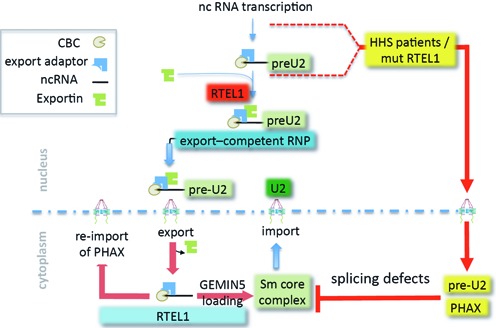
A model for RTEL1 functions in RNP trafficking. Upon transcription, nascent pre-U2 RNAs are capped and recognized by the cap-binding complex and by the protein adaptor PHAX. XPO1 interacts with RNA-bound PHAX through its NES. The production of an RNP competent for export to the cytoplasm requires RTEL1, which binds the complex in an XPO1-dependent manner; RTEL1 is also required for the correct localization of this complex in Cajal bodies. Immediately upon passing through the nuclear pore, XPO1 is released from the cargo and PHAX and CBC must be removed to allow the binding of another cap-binding protein, GEMIN5. GEMIN5 binding licenses the multistep assembly of the Sm core complex, leading to maturation of pre-U2 into U2 before the entire complex is re-imported in to the nucleus. PHAX is also re-imported into the nucleus likely through a different mechanism. Our work shows that RTEL1 governs this cytoplasmic step by negotiating the release of pre-U2 from the PHAX RNP and its loading onto GEMIN5. In the absence of RTEL1, RNA export is prevented and PHAX accumulates in the cytoplasm. Expression of RTEL1 mutants that support RNA export leads to accumulations of RNA and PHAX in the cytoplasm. The RING domain of RTEL1 appears to be absolutely required to prevent these accumulations. Export deficiency of pre-U2 in RTEL1 depleted cells, or the accumulation of cytoplasmic pre-U2 that cannot be properly utilized both lead to splicing defects.

In this work, we provide evidence showing that RTEL1 is required for the nuclear export of the pre-U2 RNP (for a model, see Figure 7). The mechanism involves a direct interaction of RTEL1 with this RNP in an XPO1-dependent manner. Whether or not RTEL1 travels with the pre-U2 RNP through the nuclear pore or just licenses it for transport remains to be determined; nevertheless, the large cytoplasmic accumulations of PHAX, the XPO1-RNP adaptor, seen both in the absence and upon expression of mutated forms of RTEL1 (as well as in RTEL1-HHS patients’ cells), provide strong evidence that RTEL1 is also exerting a crucial role in the cytoplasm. This view is supported by our experiments showing that a cytoplasmic form of RTEL1 is able to rescue such defects.

Although RTEL1 has been shown to be a helicase able to destabilize recombination DNA intermediates, we do not know whether this particular activity is relevant to the phenotypes we observe experimentally or in patients’ cells. While there is strong genetic evidence to support that this is a major activity in *Caenorhabditis elegans* (5), the non-helicase C-terminal extension of the protein, which comprises two harmonin-like domains (35), a PIP box and the cystein-rich RING domain—often affected by point mutations in HHS patients (7,10), is exclusively found in vertebrates. Our work clearly indicates that the RING domain of RTEL1, which has been so far mostly linked to genome instability phenotypes (7), plays an important role in the cytoplasmic function of the protein. Although the link between a genomic activity and RNP trafficking remains a matter of speculation, a common molecular mechanism may be invoked, possibly a ubiquitin-related protein interaction or enzymatic activity, as suggested for other proteins containing similar RING domains such as BMI1 (10,37). Interestingly, the naturally occurring shorter form of RTEL1, which is devoid of the RING domain and which is expressed at the same level as the longest isoform (10) (this report), fails to rescue the cytoplasmic defects resulting from RTEL1 deficiency. This observation is in perfect agreement with the fact that in HHS patients carrying compound heterozygous mutations in RTEL1, one of which only affects the longest isoform, the cytoplasmic phenotype is prevalent, even if they express a wild-type short isoform of the protein.

Considering the facts that RTEL1 interacts with two other major exportins and that it is not required for protein or mRNA export, we suggest that RTEL1 is part of a general mechanism for non-coding RNA trafficking. Although further studies are required to address how RTEL1 deficiencies impact microRNA trafficking (which uses XPO5) or transfer RNA export (which uses XPOT), our results, using both an experimental system and patients’ cells, indicate that RTEL1 deficiencies do have an appreciable impact on the function of the splicing machinery, most likely by perturbing the normal cell partitioning and maturation steps of RNA spliceosome components, including, but most likely not limited to, U2. Indeed, our results suggest that RTEL1 may be involved in the trafficking and biogenesis of other POLII-dependent U snRNAs such as U1 and U12, although the details remain to be explored. Thus, it is expected that such perturbations, if they are confirmed to exist in tissues of RTEL1-HHS patients, contribute to the severity of the clinical manifestations commonly described for such patients. The characterization of splicing patterns in RTEL1-HHS patients, in particular in the most affected organs such as the cerebellum, is warranted to clarify this issue.

On the other hand, given the increasingly acknowledged roles of RNPs in development, organ-specific functions and disease (38,39), other types of RNP dysfunctions may contribute to both cell proliferation defects and to the clinical manifestations in HHS. It has been shown that mouse Rtel1 is essential for embryogenesis and mouse ES cells lacking *Rtel1* die upon differentiation (1), clearly indicating that the protein is involved in functions that are not directly linked to telomere maintenance since abrogation of telomerase activity does not cause immediate embryonic lethality (40). Whether mouse Rtel1 is also involved in RNP metabolism remains to be explored.

In conclusion, our work demonstrates that it is possible to model the biological defects seen in RTEL1-HHS using an experimental system, thereby allowing the functions of RTEL1 to be further dissected and enabling us to better understand the connections between telomere biology, ncRNA RNP biogenesis, cytoplasmic processes and disease.

## SUPPLEMENTARY DATA

Supplementary Data are available at NAR Online.

SUPPLEMENTARY DATA
