# Supplementary material for: Human regulator of telomere elongation helicase 1 (RTEL1) is required for the nuclear and cytoplasmic trafficking of pre-U2 RNA
Source: Nucleic Acids Res. 2015 Jan 27;43(3):1834–47. doi: 10.1093/nar/gku1402 (PMC4330364; doi:10.1093/nar/gku1402)
Supplement: SUPPLEMENTARY DATA [file supp_43_3_1834__index.html]

Human regulator of telomere elongation helicase 1 (RTEL1) is required for the nuclear and cytoplasmic trafficking of pre-U2 RNA — SUPPLEMENTARY DATA 

# Human regulator of telomere elongation helicase 1 (RTEL1) is required for the nuclear and cytoplasmic trafficking of pre-U2 RNA

## SUPPLEMENTARY DATA

**Files in this Data Supplement:**

- Table S1
- Supplementary Figures Legends
- Supplementary Figures
