# Supplementary material for: Human regulator of telomere elongation helicase 1 (RTEL1) is required for the nuclear and cytoplasmic trafficking of pre-U2 RNA
Source: Nucleic Acids Res. 2015 Jan 27;43(3):1834–47. doi: 10.1093/nar/gku1402 (PMC4330364; doi:10.1093/nar/gku1402)
Supplement: SUPPLEMENTARY DATA [file supp_gku1402_nar-03067-a-2014-File010.docx]

*Legends to supplementary figures*

**Figure S1. Immunofluorescence experiments suggest co-localization of RTEL1 with exportins in the nucleus.**

a) Antibodies against XPO1 do not cross-react with RTEL1 protein. Protein extracts from 293T or 293T+RTEL1 cells were analyzed by Western blot using the same mouse monoclonal anti-XPO1 antibodies that were able to immunoprecipitate RTEL1. The antibodies efficiently recognize XPO1 but fail to react with RTEL1. On top, verificationof the level of expression of RTEL1 in the same extracts. b) The same anti-XPO1 antibodies are able to immunoprecipitate PHAX from HeLa cell extracts. As with RTEL1, the levels of immunoprecipitated PHAX are limited when compared to the input (5% of total). c), d), e) and f) HeLa cell preparations were pre-extracted before PFA fixation. Specific antibodies against RTEL1 and either against XPO1 (c), XPO5 (d), XPOT (e) or TRF2 (f) were revealed in either in red (RTEL1) or in green (all the others). As highlighted in the insets, signals for both RTEL1 and the exportins are reinforced at particular places within the nucleus (c,d,e), usually in proximity to the nucleolus. In contrast, such proximity of signals is nearly inexistent when using a specific marker for telomeres (TRF2, f). Bar is 5 μm.

**Figure S2. RTEL1 interacts with other U snRNAs and is not required for Cajal body formation or the export of mRNAs or of NES-containing proteins**

a) Antibodies against RTEL1 were used to assess its association with U1, U12 and U4 snRNAs by RNA-IP. Anti-RTEL1 antibodies very efficiently immunoprecipitate U1 and U12 (left histogram) and to a lesser extent U4 (right histogram), as measured by RT-qPCR. Shown is the fold enrichment compared to control IgG. b) Experiment comparing the effects of siRNAs used as controls on the relative compartmental distributions of pre-U2. Both siRNAs against Luciferase and scramble were equivalent as none of them perturbed the nuclear or cytoplasmic distributions of pre-U2, as measured by RT-qPCR, or the levels of RTEL1 or XPO1 by Western blot. In the histogram, the levels of pre-U2 were normalized to a control RNA (SGHC3) and the levels of the scramble siRNA to those of normalized Luciferase siRNA. c) IF experiments using antibodies against RTEL1 (red) and Coilin (green) (top panels) or TCAB1 (red) and Coilin (green) (bottom panels) shows normal formation of Cajal bodies in the nucleus of cells depleted for RTEL1. Cell preparations were fixed in PFA without pre-extraction (scale bar 5μm). d) RNA-FISH experiments using a poly-dT probe show unperturbed nuclear and cytoplasmic distribution of poly-adenylated mRNAs in the absence of RTEL1 (scale bar 10 mm). e) Nuclear and cytoplasmic distributions of a few chosen mRNAs in cells depleted for RTEL1 (siRTEL1) and relative to control cells transfected with an irrelevant siRNA (ctrl). Levels are normalized to an unaffected RNA (SNHG3), and normalized again to si ctrl-treated cells. f) Cells expressing a GFP protein containing a Nuclear Export Signal (NES) were transfected with either si ctrl or si RTEL1. Depletion of RTEL1 does not prevent the XPO1-dependent exportation of the protein to the cytoplasm. On the other hand, cells treated with the XPO1-inhibitor LmB do accumulate GFP in the nucleus. Cell preparations were fixed in PFA without pre-extraction.

**Figure S3. Large pre-U2 foci fail to form in RTEL1-HSS patients’ cells.**

a) Cells from a healthy control (C) or from RTEL1 HSS patients (P1 and P2) were subjected to RNA FISH with a pre-U2 probe (Figure 3a). The histogram shows the proportion of nuclei in which large pre-U2 foci were detected. More than 90 nuclei were scored for C and P2, 23 for P1. p values of pair-wise comparisons (Pearson Chi2) are indicated. b) Western blots to reveal RTEL1 in whole cell extracts from the cells examined above. Patients’ cells P1 and P2 express both RTEL1 isoforms. However, these patients are compound heterozygous (reference #10 in the main text) with one allele carrying a mutation affecting the helicase domain, present in both isoforms, and the other allele carrying a mutation affecting the RING domain, only present in the longest isoform. Thus, one of the alleles produces a wild type RTEL1_iso1_ protein.

**Figure S4. Both RTEL1 depletion and mutations lead to defects in pre-U2 RNP cytoplasmic trafficking.**

a) Levels of proteins in control and RTEL1 depleted cells upon cyclohexamide treatment. Cells depleted or not for RTEL1 were treated with 20μM cyclohexamide (CHX) for 6 hours, after which total protein extracts were prepared and analyzed with antibodies against RTEL1, SMN1 and GEMIN5. The ponceau staining is shown as loading control. b) Levels of RTEL1 proteins upon treatment of cells with siRNA (control or RTEL1) and after induction (+Tet) or not (-Tet) of RTEL1_iso6_ or RTEL1_iso6-_mR. Western blot analyzed whole cell extracts. c) A RING-mutated form of RTEL1 (mR) is unable to rescue the PHAX cytoplasmic accumulations due to depletion of RTEL1. HeLa cells carrying an inducible version of RTEL1-mR were depleted for RTEL1 and then induced or not for the expression of RTEL1_iso6_-mR. Cells were fixed with PFA without pre-extraction. Bar is 5 μm. d) Quantification of experiments in Figure 4c and S4c. More than 100 nuclei were counted for all samples, with the exception of si RTEL1+RTEL1+TET for which 50 nuclei were scored. p values of pair-wise comparisons (Pearson Chi2) are indicated. e) Expression of mutated forms of RTEL1 leads to cytoplasmic accumulations of PHAX. Quantification of the experiments shown in Figure 4d. Between 64 and 77 nuclei were scored for all samples. p values of pair-wise comparisons (Pearson Chi2) are indicated. f) Forced expression of a wild type form of RTEL1_iso6_ rescues the PHAX cytoplasmic phenotype of HHS patient’s cells. Cells from a healthy control (C) or from a HHS patient (P2) were transiently transfected with an RTEL1_iso6_ or with control vector. Cells in different conditions were analyzed by IF using antibodies against PHAX (revealed in green) and RTEL1 (revealed in red). PHAX large cytoplasmic foci are no longer detected in cells overexpressing RTEL1. Cell preparations were fixed in PFA without pre-extraction. Bar is 5 μm. Below is shown a Western blot analysis of whole cells extracts validating the overexpression of RTEL1_iso6_.g) Quantification of the above experiment. Between 57 and 84 nuclei were scored. p values of pair-wise comparisons (Pearson Chi2) are indicated.

**Figure S5. Exogenous expression of RTEL1_iso6_ carrying mutations in the NLS (mNLS) rescues the PHAX-related cytoplasmic phenotype but leads to accumulations of pre-U2 in the cytoplasm.**

a) HeLa cells expressing a nuclear form of RTEL1_iso6_-mR accumulate PHAX in the cytoplasm. These accumulations are corrected by the expression of a cytoplasmic form of RTEL1_iso6_ carrying mutations that abolish nuclear localization (mNLS) but not by RTEL1_iso1_-mNLS or RTEL1_iso6_-mR-mNLS. Quantification of the experiment shown in Figure 5c. Between 50 and 100 nuclei were scored. p values of pair-wise comparisons (Pearson Chi2) are indicated. b) Control and HSS patient’s cells were transfected transiently with vectors expressing either the long or the short isoforms of RTEL1 and carrying mutations in the NLS (mNLS). The western blot analyses shown (whole cell extracts) reveals the level of accumulation of the RTEL1 protein. PHAX and ponceau staining are included for loading control. c) RNA-FISH using a pre-U2 probe reveals accumulation of this RNA in the cytoplasm in control cells, suggesting that an intact NLS sequence in RTEL1 is necessary for correct trafficking, maturation and likely re-import of cytoplasmic pre-U2.

**Figure S6. Splicing and U snRNA accumulation defects in RTEL1-deficient cells.**

a) Cartoon illustrating the structure of the minigene used for the analysis of splicing events (reference #25). The minigene contains three exons separated by two identical (in size and sequence) introns. The smaller, middle exon can either be retained or skipped. The red arrows indicate the positions of primers used to detect the splicing products (179 and 145 bp respectively). b) Partial splicing reactions retaining the first (bottom) or second (top) introns are detected using primers overlapping exon-exon junctions. The positions of the primers are indicated by the red arrows. c) Northern blot analysis to assess the levels of POLII-dependent snRNAs U1, U2, U4 and U12 and the POLIII-dependent U6 in 293T cells or 293T cells expressing either wild type RTEL1 or the truncated form of the protein (ΔCter). The arrows on the right indicate the migration point of each species. The numbers on the right and in parenthesis represent the length in bases. d) Quantification of the signals obtained with each specific probe. Signals for U1, U2, U4 and U12 were normalized to the U6 signal in the corresponding RNA preparation. Then, ratios in 293T cells expressing either RTEL1 or ΔCter were normalized to the corresponding ratio in unperturbed 293T. While all U snRNAs tend to accumulate at higher levels in cells over-expressing the wild type protein, the levels of U1, U2 and U12 are decreased in cells expressing ΔCter. e) U snRNA-IP using anti-PHAX antibodies and nuclear extracts prepared from cells either depleted for RTEL1 or transfected with a control siRNA. The histograms show the level of enrichment with regard to IgG. In the absence of RTEL1, the interaction of PHAX with these snRNAs increases.
