# Supplementary material for: Human regulator of telomere elongation helicase 1 (RTEL1) is required for the nuclear and cytoplasmic trafficking of pre-U2 RNA
Source: Nucleic Acids Res. 2015 Jan 27;43(3):1834–47. doi: 10.1093/nar/gku1402 (PMC4330364; doi:10.1093/nar/gku1402)
Supplement: SUPPLEMENTARY DATA [file supp_gku1402_nar-03067-a-2014-File011.pptx]

## Slide 1
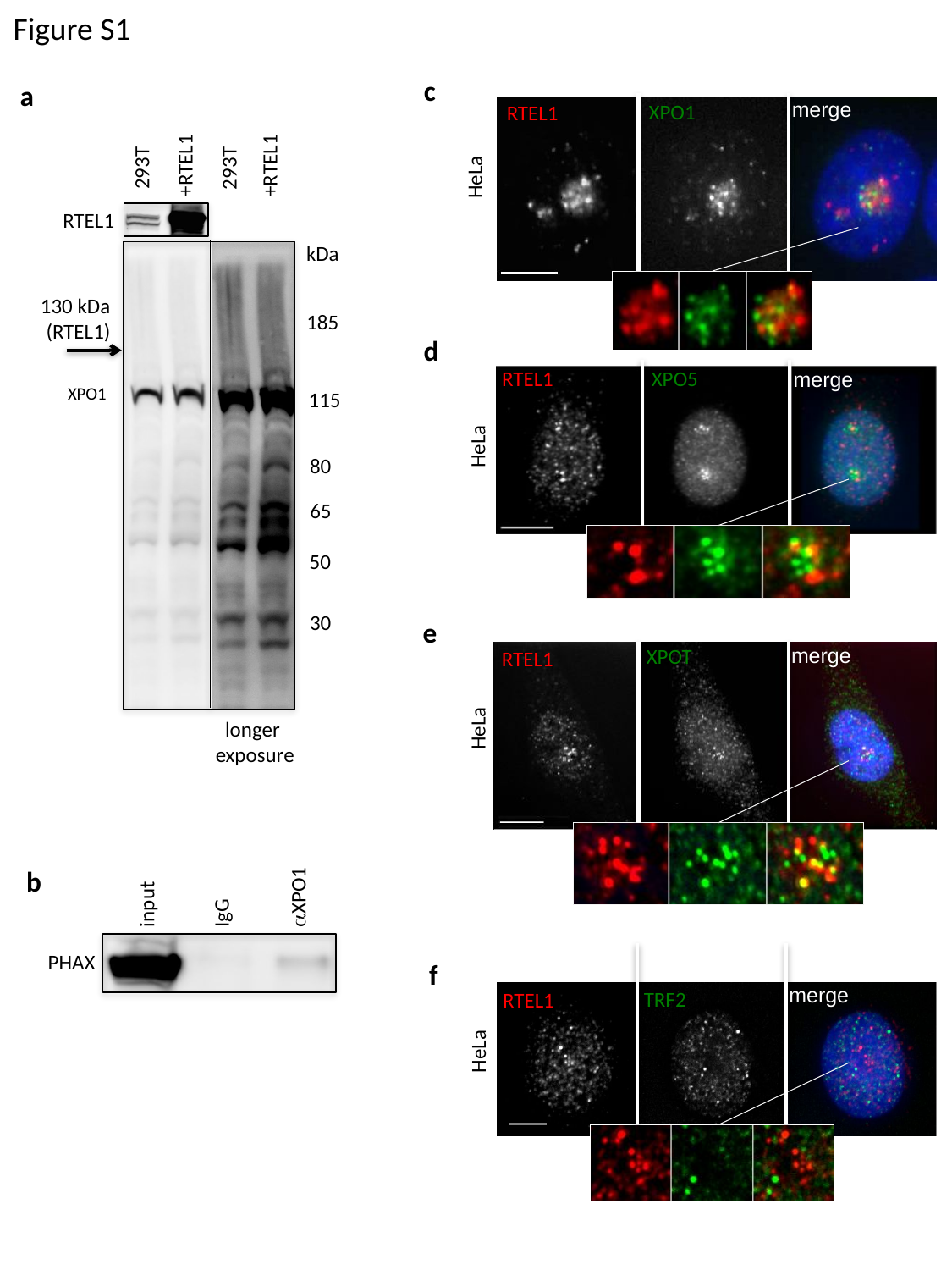

Figure S1
c
a
merge
XPO1
RTEL1
+RTEL1
+RTEL1
293T
293T
HeLa
RTEL1
kDa
130 kDa
(RTEL1)
185
d
RTEL1
XPO5
merge
HeLa
XPO1
115
80
65
50
30
e
merge
XPOT
RTEL1
HeLa
RTEL1
longer
exposure
b
aXPO1
input
IgG
PHAX
f
merge
TRF2
RTEL1
HeLa

## Slide 2
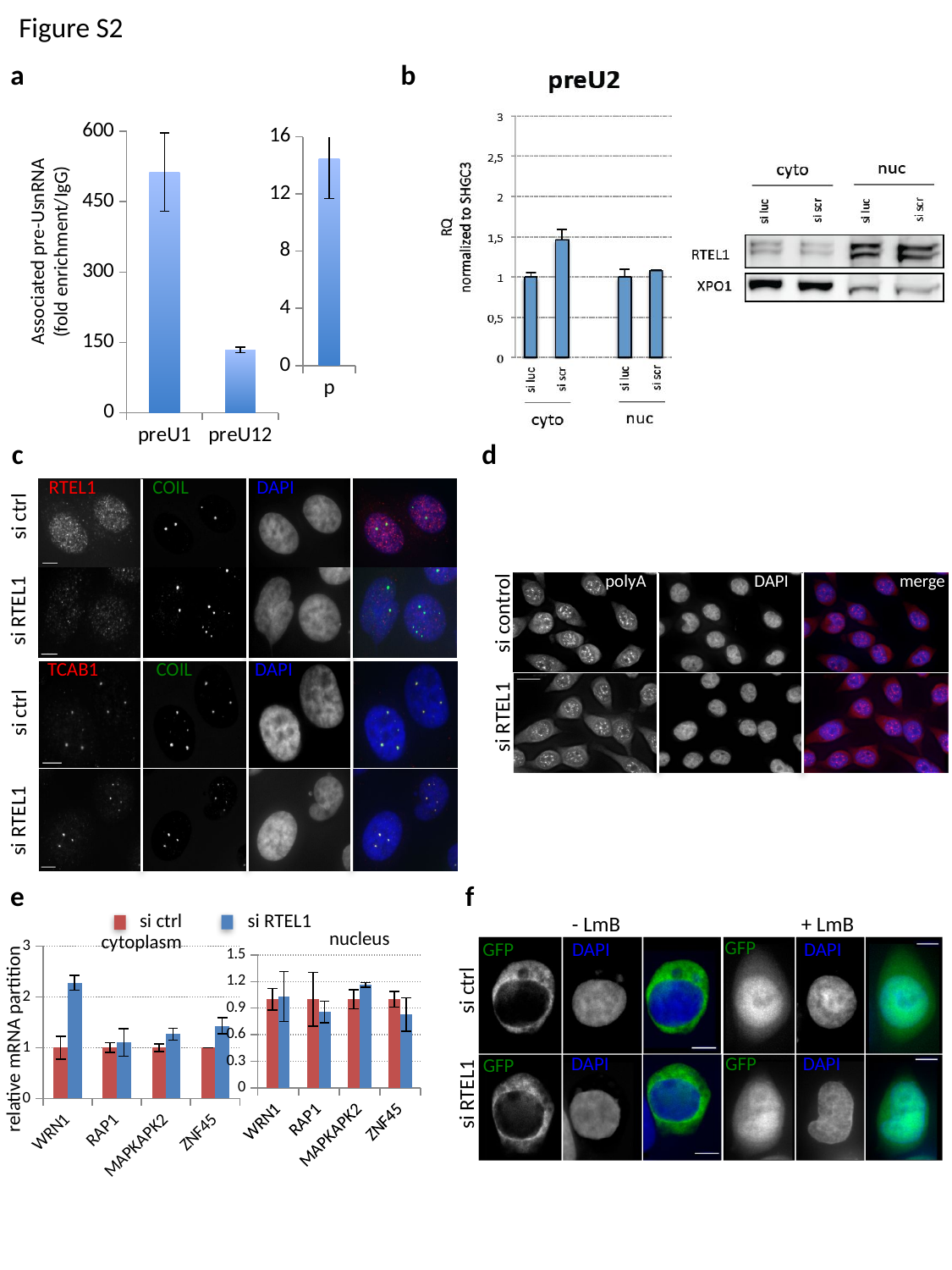

Figure S2
a
b
### Chart
| Category | RTEL1 |
|---|---|
| preU1 | 512.9939781539151 |
| preU12 | 134.314332604406 |
### Chart
| Category | RTEL1 |
|---|---|
| preU4 | 14.44277288461052 |Associated pre-UsnRNA
(fold enrichment/IgG)
c
d
COIL
DAPI
RTEL1
si ctrl
si RTEL1
COIL
DAPI
TCAB1
si ctrl
si RTEL1
polyA
DAPI
merge
si control
si RTEL1
e
f
si ctrl
si RTEL1
nucleus
### Chart
| Category | sictrl_nuc | siRTEL1_nuc |
|---|---|---|
| WRN1 | 1.0 | 1.031745977968413 |
| RAP1 | 1.0 | 0.855836901736911 |
| MAPKAPK2 | 1.0 | 1.16328338454423 |
| ZNF45 | 1.0 | 0.828168291969268 |cytoplasm
### Chart
| Category | sictrl_cyto | siRTEL1_cyto |
|---|---|---|
| WRN1 | 1.0 | 2.27311614713101 |
| RAP1 | 1.0 | 1.101110980349702 |
| MAPKAPK2 | 1.0 | 1.265877220049839 |
| ZNF45 | 1.0 | 1.430201265940397 |relative mRNA partition
+ LmB
- LmB
si ctrl
si RTEL1
GFP
GFP
DAPI
DAPI
DAPI
GFP
DAPI
GFP

## Slide 3
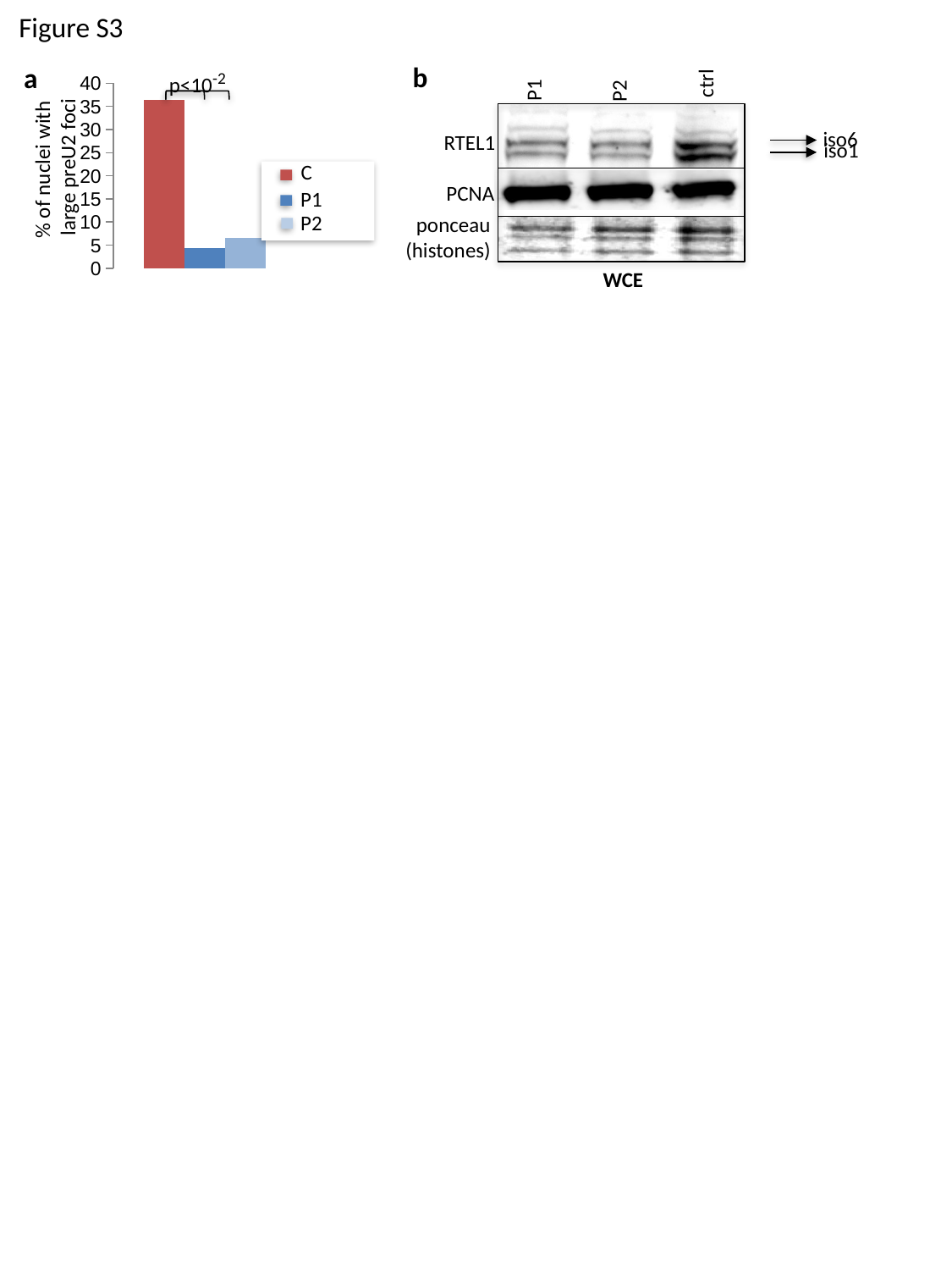

Figure S3
b
a
p<10-2
### Chart
| Category | control | P1 | P2 |
|---|---|---|---|% of nuclei with
 large preU2 foci
C
P1
P2
ctrl
P1
P2
iso6
RTEL1
iso1
PCNA
ponceau
(histones)
WCE

## Slide 4
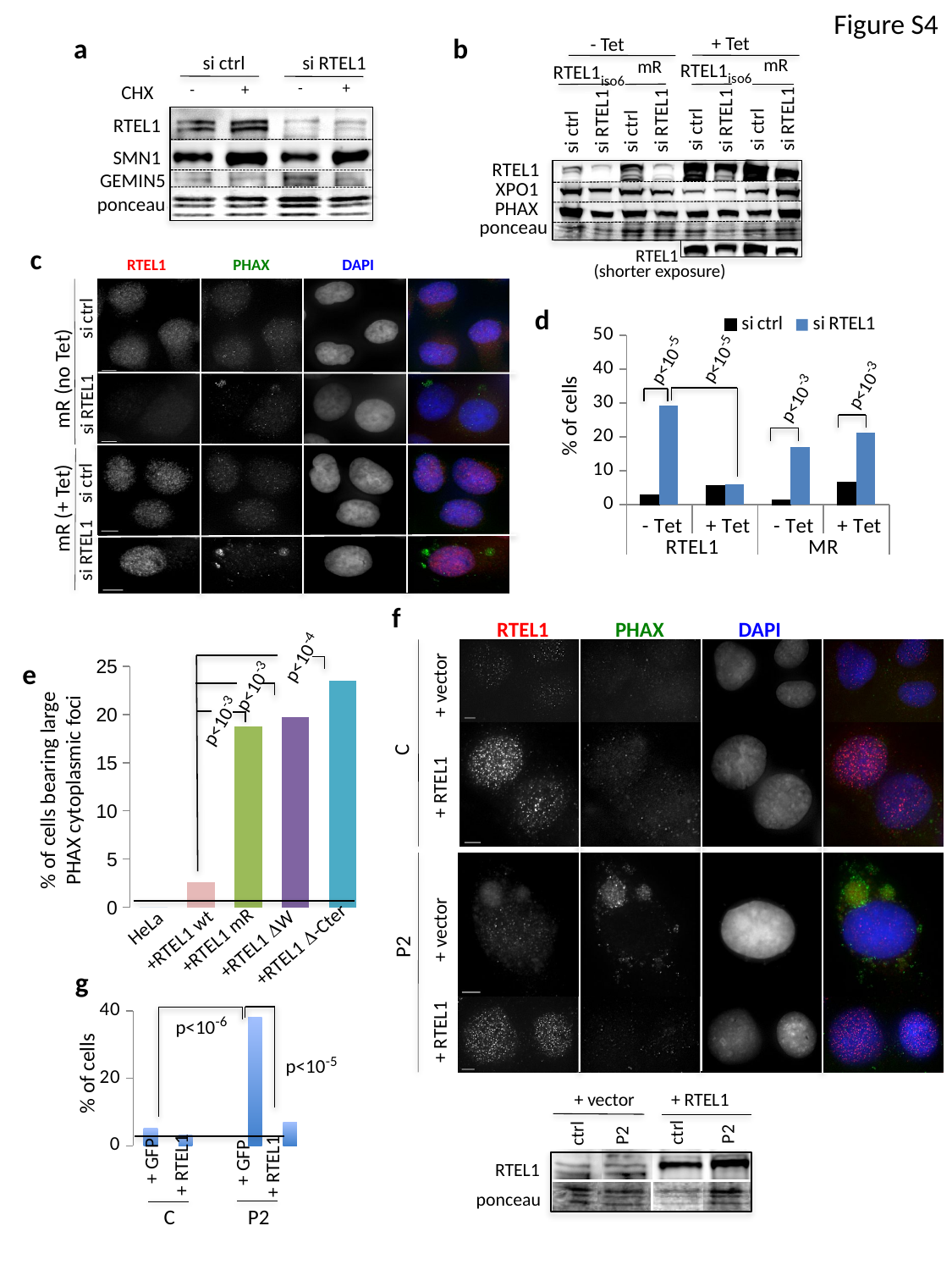

Figure S4
a
b
+ Tet
- Tet
si ctrl
si RTEL1
RTEL1iso6
mR
RTEL1iso6
mR
- +
- +
CHX
si RTEL1
si RTEL1
si RTEL1
si RTEL1
si ctrl
si ctrl
si ctrl
si ctrl
RTEL1
SMN1
RTEL1
GEMIN5
XPO1
ponceau
PHAX
ponceau
c
RTEL1
RTEL1
PHAX
DAPI
(shorter exposure)
si ctrl
d
### Chart
| Category | si ctrl | si RTEL1 |
|---|---|---|
| - Tet | 2.919708029197078 | 29.13907284768212 |
| + Tet | 5.633802816901404 | 6.0 |
| - Tet | 1.265822784810127 | 17.11711711711712 |
| + Tet | 6.56934306569343 | 21.36752136752137 |p<10-5
p<10-5
mR (no Tet)
p<10-3
p<10-3
si RTEL1
% of cells
si ctrl
mR (+ Tet)
si RTEL1
f
RTEL1
PHAX
DAPI
p<10-4
### Chart
| Category | |
|---|---|
| HeLa | 0.0 |
| RTEL1 | 2.597402597402597 |
| MR | 18.75 |
| delW | 19.73684210526316 |
| delCter | 23.52941176470588 |e
p<10-3
+ vector
p<10-3
C
% of cells bearing large PHAX cytoplasmic foci
+ RTEL1
HeLa
+ vector
+RTEL1 mR
+RTEL1 wt
+RTEL1 DW
+RTEL1 D-Cter
P2
g
### Chart
| Category | |
|---|---|
| control +GFP | 5.263157894736842 |
| control + RTEL1 | 3.278688524590164 |
| | None |
| patient 2 +GFP | 38.0952380952381 |
| patient 2 + RTEL1 | 6.896551724137931 |p<10-6
+ RTEL1
p<10-5
% of cells
+ vector
+ RTEL1
ctrl
ctrl
P2
P2
+ GFP
+ GFP
+ RTEL1
+ RTEL1
RTEL1
ponceau
P2
C

## Slide 5
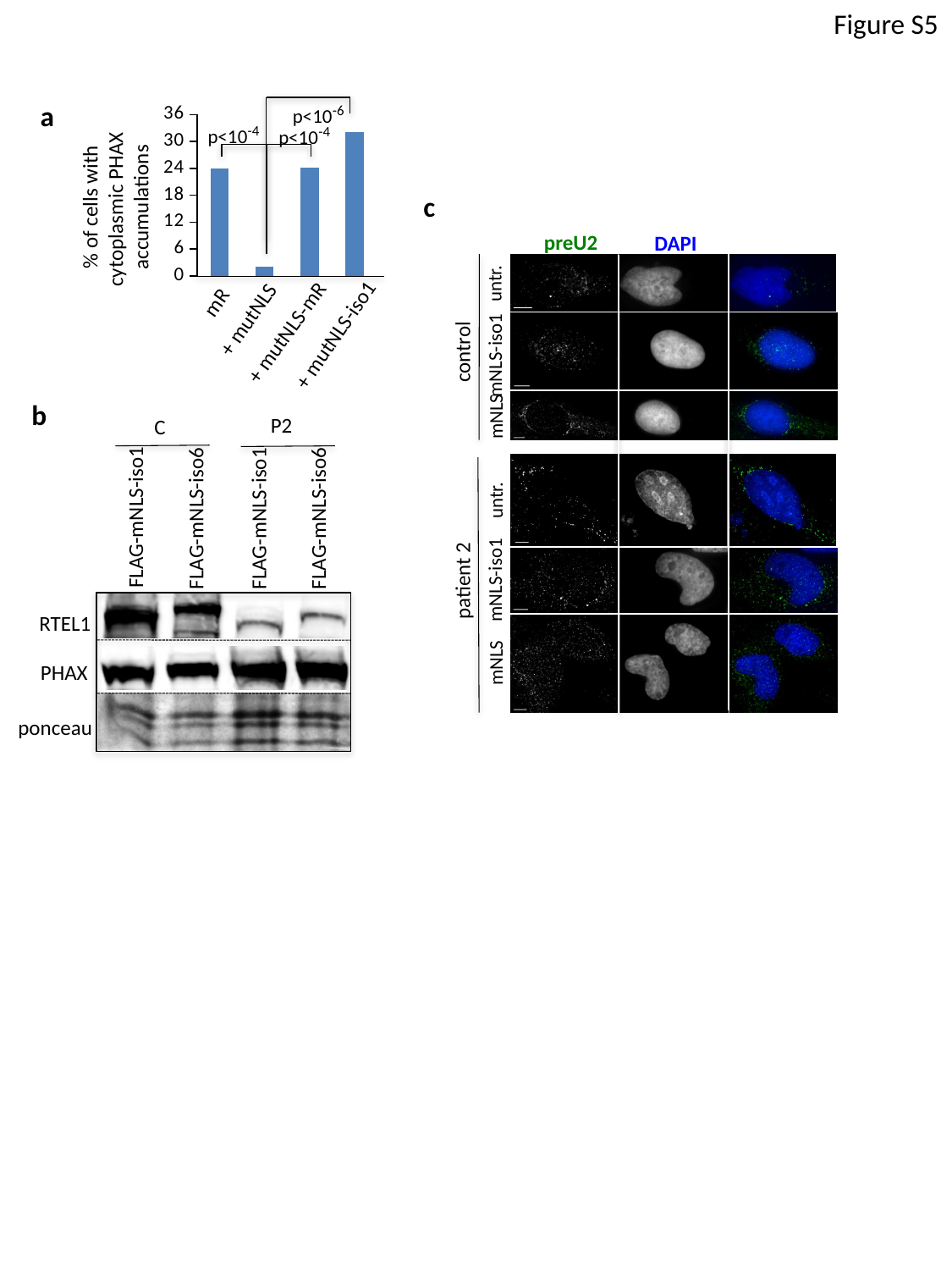

Figure S5
a
p<10-6
### Chart
| Category | PHAX |
|---|---|
| MR | 24.0 |
| NLS RTEL1 | 2.040816326530612 |
| NLS iso1 | 24.13793103448276 |
| NLS MR | 32.0 |
% of cells with cytoplasmic PHAX
accumulations
mR
+ mutNLS
+ mutNLS-mR
+ mutNLS-iso1
p<10-4
p<10-4
c
preU2
DAPI
 untr.
control
 mNLS-iso1
mNLS
 untr.
patient 2
 mNLS-iso1
mNLS
b
P2
C
FLAG-mNLS-iso1
FLAG-mNLS-iso6
FLAG-mNLS-iso6
FLAG-mNLS-iso1
RTEL1
PHAX
ponceau

## Slide 6
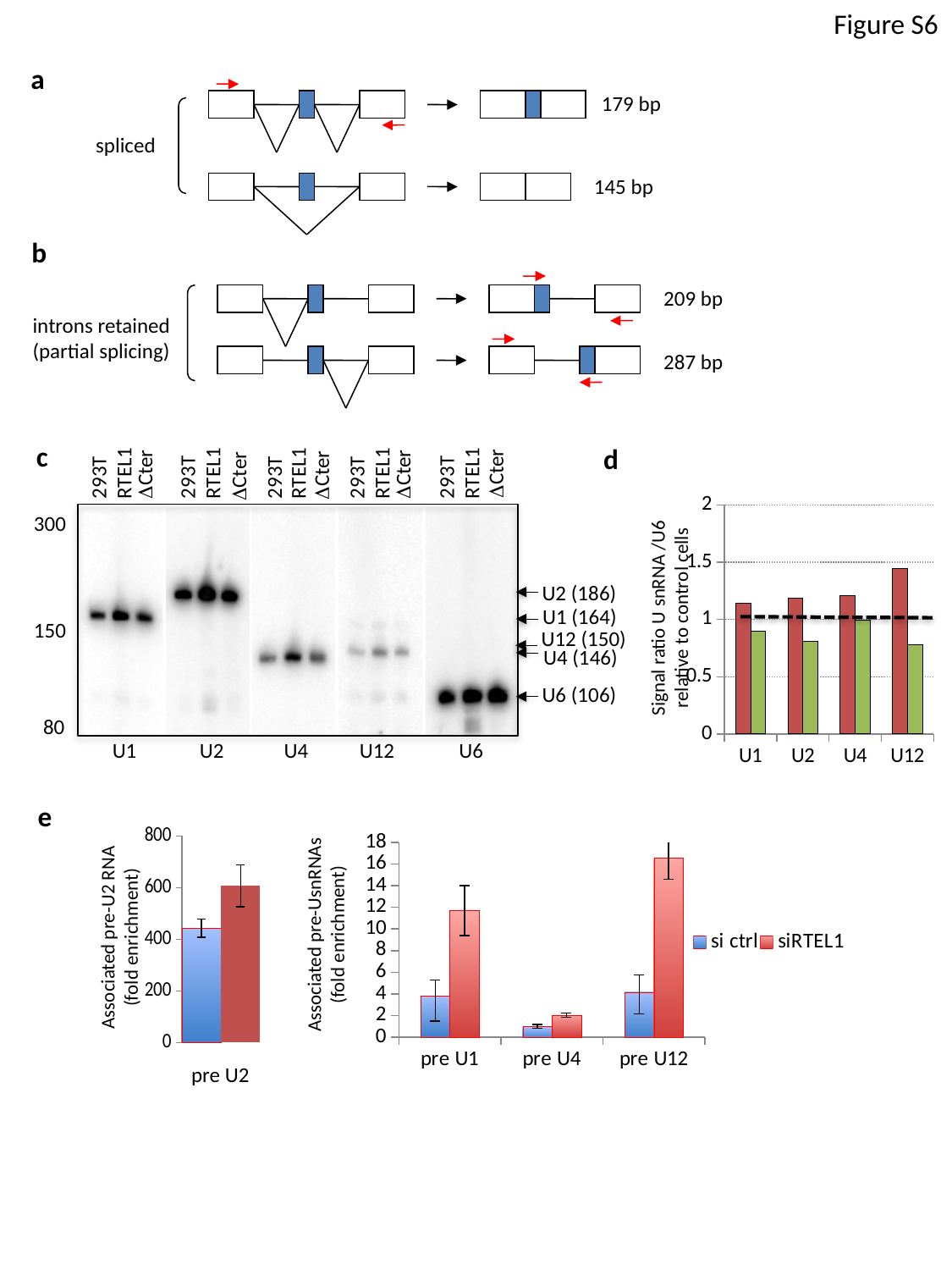

Figure S6
a
179 bp
spliced
145 bp
b
209 bp
introns retained
(partial splicing)
287 bp
c
d
DCter
DCter
RTEL1
RTEL1
RTEL1
RTEL1
RTEL1
DCter
DCter
DCter
293T
293T
293T
293T
293T
### Chart
| Category | RTEL1 | delC |
|---|---|---|
| U1 | 1.142623362813547 | 0.899922598966467 |
| U2 | 1.184181297774043 | 0.812311623372849 |
| U4 | 1.210026886941734 | 0.991822412157288 |
| U12 | 1.447673347362933 | 0.777205687892014 |300
U2 (186)
Signal ratio U snRNA /U6
relative to control cells
 U1 (164)
150
U12 (150)
U4 (146)
U6 (106)
80
U1
U2
U4
U12
U6
e
### Chart
| Category | si ctrl | siRTEL1 |
|---|---|---|
| pre U1 | 3.788921531490712 | 11.68970667567081 |
| pre U4 | 1.0 | 2.027721214415808 |
| pre U12 | 4.156651597795888 | 16.5693771161097 |
### Chart
| Category | preU2 |
|---|---|
| sictrl | 443.5812519250819 |
| siRTEL1 | 607.4436730947654 |Associated pre-UsnRNAs
(fold enrichment)
Associated pre-U2 RNA
(fold enrichment)
pre U2
